# NS3 of hepatitis C virus drives hepatocellular carcinoma progression through a novel RNA‐interference pathway

**DOI:** 10.1002/ccs3.70013

**Published:** 2025-04-12

**Authors:** Yajun Liang, Jian Luo, Liya Hu, Jun Zhang

**Affiliations:** ^1^ IV Ward of Pulmonary and Critical Care Medicine Wuhan Pulmonary Hospital Wuhan China; ^2^ Department of Geriatrics Tongji Hospital, Tongji Medical College, Huazhong University of Science and Technology Wuhan China

**Keywords:** circ_0001175, HCV NS3, hepatocellular carcinoma, miR‐130a‐5p, P53

## Abstract

Hepatocellular carcinoma (HCC), a severe consequence of hepatitis C virus infection, is significantly influenced by the virus’s non‐structural protein 3 (NS3). This study employed transcriptome sequencing to explore the role of NS3 in promoting HCC progression by comparing gene expression profiles between HCV‐infected HCC tissues and healthy liver controls. Key genes regulated by NS3 were identified and validated with quantitative reverse transcription PCR (RT‐qPCR) and western blot analyses. Functionality assays, including CCK‐8, BrdU, and Transwell migration and invasion tests, were performed to evaluate the effects of NS3 on HCC cell proliferation, migration, and invasion. Further investigation through a dual‐luciferase reporter and RNA pull‐down assays revealed that NS3 specifically upregulates circ_0001175. This circular RNA interacts with and inhibits miR‐130a‐5p, diminishing its regulatory impact on P53 by modulating the MDM4 pathway, thereby promoting oncogenic characteristics. The findings highlight the NS3‐induced circ_0001175/miR‐130a‐5p/MDM4/P53 pathway as a potential therapeutic target, offering promising directions for treatment strategies in HCV‐related HCC.

## Graphical abstract

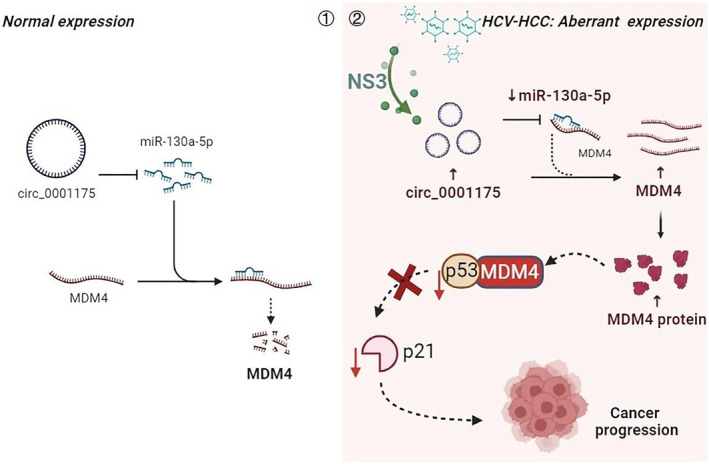

## 1 INTRODUCTION

Hepatocellular carcinoma (HCC), the leading cause of cancer‐related mortality worldwide, poses a significant global health challenge.^1^ The incidence and mortality rates of this malignancy are particularly alarming in developing countries. Infection with the hepatitis C virus (HCV) is one of the primary risk factors contributing to HCC development.^2^, ^3^, ^4^ Researchers have long been fascinated by the mechanisms through which HCV promotes the development and metastasis of liver cancer. Through further research, it has been recognized that HCV’s non‐structural protein 3 (NS3) plays a pivotal role in this particular process.^5^

HCV NS3 is essential for the viral replication cycle, playing a critical role in facilitating virus replication,^6^ suppressing antiviral responses,^7^ and interfering with interferon signaling pathways.^8^ Furthermore, NS3 has been shown to interact with p53, impairing its stability and function, thereby promoting malignant transformation in HCC cells.^9^ The protein is also associated with key signaling pathways, such as the JNK pathway^10^ and the p21/Waf1/Cip1 axis.^11^ Additionally, NS3 contributes to immune evasion, as demonstrated by studies linking it to the suppression of host immune responses.^12^ The role of noncoding Ribonucleic Acids (RNAs) in cancer progression has gained considerable attention in recent years, with molecules like Circular RNAs (circRNAs) and miRNAs emerging as critical regulators in tumorigenesis.^13^ For instance, Ying Hu et al. demonstrated that miR‐22‐based gene therapy effectively enhances antitumor immunity and metabolic processes in HCC.^14^ Similarly, Runjie Song et al. highlighted the role of cirRNA ZKSCAN1 in inhibiting mTOR signaling through the production of a cyclic peptide, shedding light on novel mechanisms in liver cancer.^15^

In recent years, advancements in transcriptome sequencing technology have provided unprecedented opportunities to unravel the complex mechanisms driving cancer development. RNA‐seq technology enables the identification of gene expression alterations at the transcriptional level and facilitates the exploration of RNA molecule interactions. This approach yields valuable insights into the processes underlying cancer initiation, progression, and metastasis. The application of transcriptome sequencing is crucial for identifying novel therapeutic targets and improving the accuracy of cancer diagnosis and prognosis.^16^, ^17^, ^18^ For example, Lei Chen and colleagues conducted a comprehensive genomic analysis of HCC in the Chinese population, revealing a genomic landscape dominated by HBV‐related HCC. Their findings provide critical insights into the evolutionary mechanisms of HCC in this population.^19^ In the present study, transcriptome sequencing technology played a key role in identifying and characterizing circ_0001175, a molecule implicated in HCV‐related HCC.

In the context of HCV infection and HCC development, the interaction between the HCV NS3 protein and circ_0001175 has emerged as a significant research focus.^20^ Prior studies have highlighted the essential role of miRNAs in this process. For instance, Liheng Li et al. demonstrated that miR‐130a‐5p suppresses liver cell proliferation, migration, and invasion induced by circ_0001175.^21^ However, the precise molecular mechanisms governing the interaction between circ_0001175 and miR‐130a‐5p remain insufficiently understood. To address this knowledge gap, researchers employ bioinformatics analyses and laboratory techniques to elucidate the functional roles of these molecules in HCV‐induced HCC progression.

MDM4 is a critical interacting protein of p53, playing a pivotal role in regulating p53’s stability and transcriptional activity. In HCC, MDM4 overexpression inhibits p53 function, thereby promoting tumor progression and contributing to poor clinical outcomes.^22^ For instance, a study conducted in eastern China reported that the GG variant of the rs1380576 locus in the MDM4 gene is associated with a reduced risk of liver cancer, particularly in elderly males, nonsmokers, and nondrinkers.^22^ These findings suggest that genetic variations in MDM4 could serve as potential biomarkers for liver cancer susceptibility. Additionally, splice variants of MDM4 may influence its function and stability, affecting the biological behavior of liver cancer cells. Ongoing research is investigating the roles of these splice isoforms in liver cancer development to evaluate their potential as therapeutic targets or biomarkers.^23^

The objective of this study is to investigate, using transcriptome sequencing, how the HCV NS3 protein contributes to the malignant progression of HCV‐related HCC through the circ_0001175/miR‐130a‐5p/MDM4/P53 pathway. This research aims to deepen our understanding of the molecular mechanisms underlying HCC while providing novel insights and strategies for its prevention and treatment. The findings hold significant scientific and clinical implications, offering promising avenues for developing targeted therapies and improving the prognosis of patients with HCV‐induced HCC.

## 2 MATERIALS AND METHODS

### 2.1 This study focused on high‐throughput sequencing of transcriptome samples, including data quality control and differential gene analysis

Total RNA was extracted from HCCLM3 cells overexpressing NS3 and control cells using TRIzol reagent (Thermo, 16096020, USA) in triplicate (*n* = 3). RNA concentration was measured using a Qubit® 2.0 Fluorometer (Life Technologies, Q33216, USA) with the Qubit® RNA Analysis Kit (Shanghai Boji Biotechnology, HKR2106‐01, China) and a Nanodrop spectrophotometer (IMPLEN, USA). RNA purity was confirmed by the A260/A280 ratio (1.8–2.0), and integrity was assessed with the RNA Nano 6000 assay kit (Agilent, 5067‐1511, USA) on a Bioanalyzer 2100 system (Agilent, USA). RNA quality met the required standards for reliable experimental results.

For library preparation, 3 μg of total RNA per sample was processed using the NEBNext® Ultra™ RNA Library Prep Kit (NEB, E7435 L, China) for Illumina® sequencing. Library quality was evaluated with a Bioanalyzer 2100 system. Index‐coded samples were clustered on the cBot system using the TruSeq PE Cluster Kit v3 cBot HS (Illumina, PE‐401‐3001, USA) and sequenced on the Illumina HiSeq 550 platform, generating 125 bp or 150 bp paired‐end reads.

Raw sequencing data quality was evaluated with FastQC (v0.11.8). Data preprocessing was performed using Cutadapt (v1.18) to remove adapters and poly(A) tails. Reads with >5% N content were removed using a Perl script, and those with ≥70% bases of Q20 quality were retained using the FASTX Toolkit (v0.0.13). Paired‐end sequences were repaired with BBMap software, and high‐quality reads were aligned to the human reference genome using HISAT2 (v0.7.12). Differential gene expression between HCCLM3‐vector and HCCLM3‐NS3 samples was analyzed using the “Limma” package in R, with thresholds of |logFC| > 1 and *p* < 0.05. Heatmaps and volcano plots were generated using the “heatmap” and “ggplot2” packages in R, respectively.^24^, ^25^, ^26^, ^27^

For circRNA analysis, total RNA was treated with RNase R (20 U/μL, Epicentre, RNR07250, USA) to remove linear RNA and enrich circRNAs. Amplification and transcription were performed with the Low Input QuickAmp Labeling Kit One‐Color (Arraystar, 5190‐2305, USA). cRNAs were hybridized to the Human circRNA Array (AS‐S‐CR‐H‐V2.0, 8 × 15 K, Arraystar, USA) and incubated at 65°C for 17 h in a SHELLAB1013‐2 hybridization oven. Volcano plots and heatmaps were used to identify circRNAs with fold changes ≥ 2 and *p*  ≤  0.05 between HCCLM3‐vector and HCCLM3‐NS3 samples.^28^

### 2.2 Bioinformatics analysis

The binding sites of circRNA and miRNA were predicted using the StarBase database (https://starbase.sysu.edu.cn/). Utilize the Venn Diagram tool for Venn analysis to identify candidate genes.^29^

### 2.3 Clinical sample collection

Tumor specimens were collected from 46 HCV‐positive HCC patients (*n* = 46) and 46 HCV‐negative HCC patients (*n* = 46) at our hospital. All specimens were derived from the primary tumors of liver cancer patients. There are 50 male and 42 female participants, with an average age of 57.8 years. None of the patients received chemotherapy, radiotherapy, or any other special treatment before the surgery. The collected sample is divided into two parts: one part is promptly preserved in liquid nitrogen, whereas the other part is fixed in 10% formaldehyde and embedded in paraffin for subsequent sectioning. This study has received approval from the Clinical Ethics Committee of our institute and has obtained informed consent from the patients in strict adherence to the requirements of the Helsinki Declaration. Detailed information for each patient is presented in Table S1.^30^

### 2.4 Cell culture, transfection, and sorting

HCCLM3 cells (bio‐73219, Biobw, China) and Huh‐7 cells (MZ‐1984, Ningbo Mingzhou Biological Technology Co., Ltd., China) were cultured in RPMI 1640 complete medium (R4130, Sigma, USA) supplemented with 10% fetal bovine serum (F8318, Sigma, USA), 100 U/mL penicillin, and 100 μg/ml streptomycin (V900929, Sigma, USA). The cells were maintained at 37°C in a humidified incubator with 5% CO_2_.

Plasmids and lentiviruses were synthesized by Gima Shanghai Company. The NS3 gene sequence (GenBank: ON951661.1) was obtained from NCBI. The constructed pHAGE‐puro plasmids, along with helper plasmids pSPAX2 and pMD2. G, were co‐transfected into 293T cells. Viral supernatants were collected at 48 and 72 h, filtered through a 0.45 μm filter, and concentrated by centrifugation. HCCLM3 and Huh‐7 cells were seeded at 5 × 10^4^ cells/mL into 6‐well plates and infected after reaching 70%–80% confluency. Stable cell lines were established by culturing infected cells in a complete medium containing 2 mg/ml puromycin for 5 days.

Western blot was used to detect the expression levels of the relevant genes in the cell groups. The shRNA sequences were designed using Life Technologies and established stable passaged transfectant cell lines named HCCLM3‐NS3 and Huh‐7‐NS3 (referred to as NS3T, indicating cells capable of stably expressing NS3). HCCLM3 and Huh‐7 cells were also transfected with empty plasmids, referred to as HCCLM3‐vector and Huh‐7‐vector cells (referred to as UT, indicating cells transfected with empty vectors, serving as negative controls).

ShRNA targeting circ_0001175 (sh‐circ_0001175#1 and sh‐circ_0001175#2) and the negative control sh‐NC, miR‐130a‐5p mimic (miR‐130a‐5p) and anti‐miR‐130a‐5p, along with their corresponding negative controls (miR‐NC and anti‐NC), and MDM4 overexpression vector (oe‐MDM4) and matched controls were obtained from Ribobio, China. Similarly, these substances were transfected into UT and NS3T cells using lentiviral transduction. Refer to Table S2 for interference sequences.

When preparing the cell transfection for the construction of the lung metastasis model, transfection began when HCCLM3‐NS3 and Huh‐7‐NS3 cells reached 70%–80% density. Following the manufacturer’s instructions, plasmids containing the firefly luciferase gene were mixed with transfection reagents using Lipofectamine 3000 (L3000150, Thermo Fisher) and added to the culture dishes of HCCLM3‐NS3 and Huh‐7‐NS3 cells. The cells were then continuously cultured at 37°C with 5% CO_2_ in a cell culture incubator for 24–48 h. At 24–48 h post transfection, a small number of cells was transferred to a 96‐well plate, appropriate D‐luciferin (L2916, Thermo Fisher) was added to each well, and a tabletop luminometer was used to detect the light signal to verify luciferase expression. The groups were divided as follows: 1) UT (HCCLM3‐vector/Huh‐7‐vector); 2) NS3T (HCCLM3‐NS3/Huh‐7‐NS3); 3) NS3T + sh‐NC + oe‐NC (HCCLM3‐NS3T/Huh‐7‐NS3+sh‐NC + oe‐NC); 4) NS3T + sh‐circ_0001175#1+oe‐NC (HCCLM3‐NS3T/Huh‐7‐NS3+sh‐circ_0001175#1+oe‐NC); and 5) NS3T + sh‐circ_0001175#1+oe‐MDM4 (HCCLM3‐NS3T/Huh‐7‐NS3+sh‐circ_0001175#1+oe‐MDM4).^31^, ^32^

Furthermore, when investigating the expression of miR‐130a‐5p, cell groups were constructed by lentiviral transduction with HCCLM3‐NS3 and Huh‐7 cells as follows: sh‐NC group, sh‐circ_0001175#1 group, sh‐circ_0001175#1 + anti‐NC group, and sh‐circ_0001175#1 + anti‐miR‐130a‐5p. Similarly, in exploring MDM4‐related expression, cell groups were constructed by lentiviral transduction with HCCLM3‐NS3 and Huh‐7 as follows: miR‐NC, miR‐130a‐5p, miR‐130a‐5p + oe‐NC, and miR‐130a‐5p + oe‐MDM4.

### 2.5 Reverse transcription quantitative polymerase chain reaction (RT‐qPCR)

Total RNA was extracted using TRIzol (15596026, Thermo Fisher, USA), and concentration and purity were assessed with a NanoDrop 2000 spectrophotometer (Thermo Fisher, USA). Reverse transcription of mRNA was performed using the PrimeScript RT Reagent Kit (RR047 A, Takara, Japan), and miRNA reverse transcription was carried out with the TaqMan MicroRNA Assays (4427975, Thermo Fisher, USA).

RT‐qPCR was conducted using the Fast SYBR Green PCR kit (11736059, Thermo Fisher, USA) in triplicate. Glyceraldehyde‐3‐phosphate dehydrogenase (GAPDH) was used as the reference for mRNA and circRNA, whereas U6 served as the reference for miRNA. Relative gene expression was calculated using the 2^−ΔΔCt^ method, with ΔCt representing the difference between target and reference genes. Primer sequences are detailed in Table S3. All experiments were repeated three times.^33^

### 2.6 Western Blot

Proteins were extracted using Radioimmunoprecipitation Assay (RIPA) lysis buffer (P0013 B, Beyotime Biotechnology, China), and concentrations were quantified with a BCA assay kit (A53226, Thermo Fisher, USA). Proteins were separated via Sodium Dodecyl Sulfate‐Polyacrylamide Gel Electrophoresis and transferred onto Polyvinylidene Fluoride membranes (IPVH85 R, Millipore, Germany) using the wet transfer method. Membranes were blocked with 5% Bovine Serum Albumin for 1 h at room temperature and then incubated overnight at 4°C with primary antibodies (Table S4).

After washing with Tris‐Buffered Saline with Tween‐20, membranes were incubated for 1 h with HRP‐conjugated goat anti‐rabbit IgG (ab97051, Abcam, UK) or goat anti‐mouse IgG (ab205719, Abcam, UK), both diluted 1:2000. Protein bands were visualized using an Enhanced Chemiluminescence detection kit (abs920, GeneCopoeia, China) and analyzed with Bio‐Rad Quantity One V4.6.2 software. GAPDH served as the loading control. Each experiment was repeated three times, and the results were averaged.^34^

### 2.7 CircRNA validation

To confirm that circ_0001175 is a circRNA, reverse transcription was performed using Oligo(dT)18 primers (SO132, Thermo Fisher, USA) and Random primers (48190011, Thermo Fisher, USA). Oligo(dT)18 primers hybridize with the 3’‐polyA tails of linear RNAs but cannot hybridize with circRNAs, which lack a 3’ tail. Expression levels of circ_0001175 were determined using RT‐qPCR.^30^

### 2.8 Cellular localization analysis

Total RNA was extracted from the cytoplasm and nucleus using the PARIS™ Kit (AM1921, Thermo Fisher, USA). The expression levels of circ_0001175, U6, and GAPDH were measured by RT‐qPCR. U6 and GAPDH were used as reference genes for the nucleus and cytoplasm, respectively.^30^

### 2.9 CCK‐8

Cell viability was assessed using the CCK‐8 assay kit (ab228554, Abcam, USA). Cells (2500 per well) were seeded into 96‐well plates and cultured for 24, 48, 72, and 96 h. At each time point, 10 μL of CCK‐8 reagent was added to each well, followed by incubation for 2 h. Absorbance was measured at 450 nm using a Multiskan Flow Cytometry ELISA reader (51119080, Thermo Fisher, USA).^35^

### 2.10 BrdU cell proliferation assay

HCCLM3‐SN3 and Huh‐7‐SN3 cells in the logarithmic growth phase were prepared as single‐cell suspension and seeded into a 24‐well plate (1 × 10^5^ cells per well) with BrdU staining reagent (ST1056, Beyotime, Shanghai, China). After 12 h of incubation, cells were fixed with 4% formaldehyde and incubated with anti‐BrdU antibody (ab308341, 1:2000, Abcam, USA) and 4′,6‐diamidino‐2‐phenylindole (DAPI) (C1006, Beyotime, Shanghai, China) for a staining solution. BrdU‐positive and DAPI‐positive cells were counted in three fields of view using a fluorescence microscope (Olympus, Tokyo, Japan). The proliferation rate was calculated as the ratio of BrdU‐positive cells to DAPI‐positive cells.^21^

### 2.11 Transwell migration and invasion assays

To analyze cell migration ability, 2 × 10^4^ transfected HCCLM3‐NS3 and Huh‐7‐NS3 cells were suspended in 200 μL of a serum‐free RPMI 1640 medium and then added to the upper chamber of a Transwell without pre‐coated Matrigel (356234, BD, USA).

To analyze cell invasion ability, Matrigel (356234, BD, USA) was diluted in a serum‐free RPMI 1640 medium at a ratio of 1:10. Subsequently, 100 μL of the diluted Matrigel was added to the upper chamber of the Transwell and incubated for a minimum of 30 min. Following the incubation period, cells were seeded into the Matrigel‐coated upper chamber.

In both assays, 600 μL of 10% FBS‐containing 1640 medium was added to the lower chamber. The chambers were then incubated at 37°C in a CO_2_ incubator for 24 h. Afterward, the cells were fixed with 4% paraformaldehyde for 15 min, stained with 0.1% crystal violet for 15 min, and subsequently observed for positively stained cells using an inverted light microscope (Carl Zeiss, Germany). Finally, the cells were photographed. Positive cells were counted using ImageJ software.^36^

### 2.12 RNA pull‐down assay

HCCLM3‐NS3 and Huh‐7‐NS3 cells were lysed with RIPA lysis buffer (89901, Thermo Fisher, USA). Subsequently, miR‐130a‐5p was labeled using the Biotin RNA Labeling Kit (11685597910, Roche, China) according to the manufacturer’s instructions to obtain Bio‐NC, Bio‐miR‐130a‐5p, and Bio‐miR‐130a‐5p mutant (MUT). Dynabeads® M‐280 streptavidin (11206D, Thermo Fisher, USA) was subsequently used for isolating biotin‐labeled RNA. Then, RT‐qPCR was performed to assess the abundance levels of circ_0001175 and MDM4 mRNA.^21^

### 2.13 Dual‐luciferase reporter assay

The circ_00011754/MDM4 Wild Type (WT) or circ_00011754/MDM4 3’UTR MUT sequences containing the miR‐130a‐5p binding sites were cloned into the pGL3‐basic vector (HG‐VQP0121, Promega Corporation, USA). The plasmids carrying the firefly luciferase reporter and either miR‐NC or miR‐130a‐5p were transfected into cells plated in a 48‐well plate (8 × 10^3^ cells/well) using Lipofectamine 3000 (Invitrogen). After 48 h of transfection, the relative luciferase activity was measured using the dual‐luciferase reporter assay kit (D0010, Solarbio, China), with Renilla luciferase activity serving as the control.^30^

### 2.14 Build mouse model

6‐week‐old female BALB/c nude mice (strain code: 401) were obtained from Beijing Vital River Laboratory Animal Technology Co., Ltd. The mice were individually housed in separate cages within the Specific Pathogen Free grade animal laboratory. The humidity in the facility is maintained at 60%–65%, and the temperature ranges from 22 to 25 degrees Celsius. After 1 week of adaptive feeding, the mice’s health status was observed before the commencement of the experiment. The experimental procedure and animal usage plan have been approved by the Animal Ethics Committee.

To establish a subcutaneous xenograft model in mice, processed liver cancer cells (1.0 × 10^6^) were injected subcutaneously. Tumor size was measured weekly, and mice were grouped as follows: (1) control group (HCCLM3 cells transfected with empty vector), (2) NS3T group (cells transfected with pHAGE‐NS3), (3) NS3T + sh‐NC + oe‐NC group, (4) NS3T + sh‐circ_0001175+oe‐NC group, and (5) NS3T + sh‐circ_0001175+oe‐MDM4 group. Tumors were harvested 4 weeks after inoculation for RT‐qPCR and immunohistochemistry to analyze the expression of circ_0001175, miR‐130a‐5p, MDM4, P53, and P21.

To construct a lung metastasis model in nude mice, 1 × 10^6^ cells were injected via the lateral tail vein into groups of 6 mice. Luminescent signals were analyzed using the CRi Maestro in vivo imaging system (CRi Inc., USA). After 8 weeks, mice were euthanized and lung tissues were fixed, stained, and analyzed for metastatic nodules.^30^, ^37^

### 2.15 H&E staining

Tumor tissues were fixed in 10% neutral formalin, embedded in paraffin, and sectioned. Sections were deparaffinized, stained with hematoxylin and eosin, dehydrated, and mounted with neutral resin. Samples were observed under an optical microscope for histological analysis.^38^

### 2.16 Immunohistochemistry

The tissue specimens were immersed in 4% paraformaldehyde for 12 h, followed by the preparation of paraffin sections with a thickness of 3 μm. Standard xylene dewaxing was performed, followed by gradient ethanol hydration using anhydrous ethanol, 95% ethanol, and 75% ethanol for 3 min each. For antigen retrieval, the sections were immersed in 0.01 M citrate buffer, heated to boiling for 15–20 min, and allowed to cool at room temperature for 30 min to deactivate endogenous peroxidase activity.

Blocking was carried out by incubating the sections with goat serum‐blocking solution at room temperature for 20 min, after which excess liquid was removed. Next, 50 μL of Ki‐67 antibody (catalog number ab15580, diluted 1:100, Abcam, USA) was added and incubated at room temperature for 1 h, followed by Phosphate Buffered Saline (PBS) washing. Subsequently, an anti‐mouse IgG goat antibody (catalog number ab205719, diluted 1:10000, Abcam, UK) was applied and incubated at 37°C for 1 h. After washing with PBS, streptavidin‐peroxidase (SP) was added, and the sections were incubated at 37°C for 30 min before another PBS wash.

3,3'‐diaminobenzidine reagent (catalog number ST033, Guangzhou Wei Jia Technology Co., Ltd., China) was used for color development, which lasted 5–10 min and was stopped by washing with water for 10 min. Counterstaining was performed using PT001 (Shanghai Bogu Biotechnology Co., Ltd., China), followed by differentiation with hydrochloric acid alcohol and rinsing in water for 10 min. Sections were dehydrated using an ethanol gradient, cleared with xylene, and embedded in neutral resin.

Observation and analysis were conducted using an upright microscope (model BX63, Olympus, Japan). Five high‐magnification fields of view were randomly selected for each slide, and the average light density of the images was analyzed using Image‐Pro Plus 6.0 software. The experiment was repeated three times. Positive staining was assessed under a light microscope.^39^, ^40^

### 2.17 Statistical analysis

Statistical analyses were conducted using GraphPad Prism version 9.0 (GraphPad Software, San Diego, CA, USA). Data are expressed as mean ± standard deviation, and all experiments were independently repeated at least three times. Statistical significance was set at *p* < 0.05. Two‐group comparisons were performed using independent two‐sample *t*‐tests, with assumptions of normality and homogeneity of variance validated prior to analysis. For comparisons involving more than two groups, one‐way analysis of variance (ANOVA) was utilized, followed by Tukey’s post hoc test for pairwise comparisons. Time‐dependent cellular activities measured across multiple time points and experimental conditions were analyzed using two‐way repeated‐measures ANOVA, with Bonferroni correction applied for post hoc tests where necessary. Pearson's correlation coefficient was used to examine linear relationships between continuous variables. For tumor growth data in the nude mouse xenograft model, repeated‐measures ANOVA was employed to evaluate differences in tumor volume over time among groups. All analyses were performed with appropriate tests to ensure the robustness and reliability of the findings.

## 3 RESULTS

### 3.1 The HCV NS3 protein promotes the proliferation, migration, and invasion of liver cancer cells

Prior studies have shown that HCV infection induces Epithelial‐Mesenchymal Transition (EMT) in cultured cells, enhancing cellular motility and invasiveness.^41^ Moreover, HCV facilitates liver cancer progression and metastasis by upregulating NS3 protein expression, which augments the invasive capacity of HCV‐infected liver cancer cells.^42^

**FIGURE 1:**
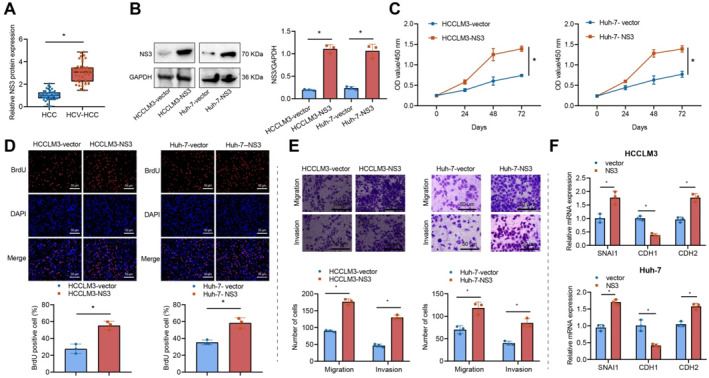
Impact of hepatitis C virus (HCV) NS3 on HCC cell proliferation, migration, and invasion. *Note:* (A) NS3 protein expression in HCV‐positive and HCV‐negative patients, detected by the western blot (*n* = 40). (B) NS3 protein expression in HCCLM3‐vector, HCCLM3‐NS3, Huh‐7‐vector, and Huh‐7‐NS3 cell lysates, examined by the western blot. (C–D) Regulation of HCCLM3 and Huh‐7 cell proliferation by HCV NS3 protein, assessed using CCK‐8 and BrdU assays. (E) Regulation of HCCLM3 and Huh‐7 cell migration and invasion by HCV NS3 protein, evaluated by Transwell assay. (F) SNAI1, CDH1, and CDH2 mRNA levels in HCCLM3 and Huh‐7 cells, determined by RT‐qPCR. * indicates a difference between the two groups (*p* < 0.05). All cell experiments were repeated three times (*n* = 3). HCC, Hepatocellular carcinoma; NS3, Non‐structural protein 3; RT‐qPCR, Reverse Transcription Quantitative Polymerase Chain Reaction.

To investigate the role of HCV NS3 in liver cancer, we analyzed NS3 protein levels in HCV‐positive and HCV‐negative patient samples using western blotting. The results revealed a significant increase in NS3 expression in HCV‐positive patients (Figure 1A). To enhance our comprehension of how NS3 impacts liver cancer cells, we initially developed two human HCC cell lines with a stable expression of NS3 protein (HCCLM3‐NS3 and Huh‐7‐NS3). The stable HCCLM3 and Huh‐7 transfectants exhibited HCV NS3 protein expression (Figure 1B). Results from the CCK‐8 and BrdU experiments demonstrated an increase in liver cancer cell proliferation in both NS3 overexpression groups compared to the control group (Figure 1C–D). The Transwell results demonstrated that the NS3 protein enhanced the migration and invasion of two types of liver cancer cells (Figure 1E). SNAI1, CDH1, and CDH2 are commonly employed as crucial molecular markers in EMT research.^43^ The results from the RT‐qPCR analysis revealed that NS3 increased the mRNA levels of SNAI1 and CDH2 while reducing the expression of CDH1 (Figure 1F). The above results suggest that the HCV NS3 protein enhances the proliferation, migration, and invasion of HCCLM3 and Huh‐7 liver cancer cells.^21^

### 3.2 HCV NS3 protein induces overexpression of circ_0001175 in liver cancer cells

**FIGURE 2:**
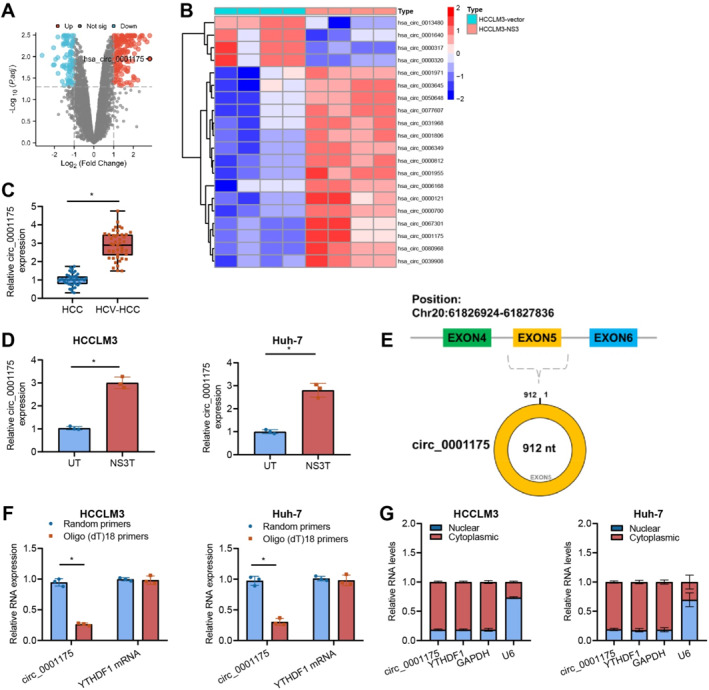
Differential expression of circ_0001175 in HCV‐positive HCC tissues and cell lines. *Note:* (A–B) Microarray analysis of circ_0001175 expression in HCCLM3‐NS3 cells versus HCCLM3‐vector cells, shown through (A) volcano plot and (B) heatmap analysis. (C) Expression of circ_0001175 in HCV‐negative and HCV‐positive HCC tissues (*n* = 46) by RT‐qPCR. (D) Expression of circ_0001175 in HCCLM3‐NS3 and Huh‐7‐NS3 cell lines by RT‐qPCR. (E) Structure of circ_0001175. (F) Verification of circRNAs using Oligo(dT)18 primers. (G) Determination of the cellular distribution of circ_0001175 using RT‐qPCR. * indicates a difference between the two groups (*p* < 0.05). All cell experiments were repeated three times (*n* = 3). circRNA, Circular RNA; HCC, Hepatocellular carcinoma; NS3, Non‐structural protein 3; RT‐qPCR, Reverse Transcription Quantitative Polymerase Chain Reaction.

CircRNAs have been demonstrated to play a vital role in numerous tumor types, including HCC.^44^ To evaluate the involvement of circRNAs in HCV‐positive liver cancer, microarray analysis was performed on HCCLM3‐NS3 and HCCLM3‐vector cells. A volcano plot illustrated the differential expression of circRNAs (Figure 2A). Among the top 25 differentially expressed circRNAs, circ_0001175 showed significant upregulation, which was further explored using a heatmap (Figure 2B).

The results of the RT‐qPCR experiment revealed an increase in the expression level of circ_0001175 in HCV‐positive liver cancer tissues (Figure 2C). Furthermore, the expression of circ_0001175 was increased in both HCCLM3‐NS3 and Huh‐7‐NS3 liver cancer cell lines, as illustrated in Figure 2D. The circ_0001175 RNA molecule is produced from exon 5 of the YTHDF1 gene, measuring 912 base pairs in length (Figure 2E). To further validate the characteristics of circ_0001175, we utilized Oligo(dT)18 primers for verification. The primer specifically binds to linear mRNA, not cirRNA. Therefore, circ_0001175, with its circular structure, cannot be detected using the Oligo(dT)18 primer (Figure 2F). Furthermore, subcellular fractionation experiments have demonstrated that circ_0001175 is predominantly localized in the cytoplasm (Figure 2G).

In conclusion, the HCV NS3 protein could cause an increase in the expression of circ_0001175 in cells.

### 3.3 Knockdown of circ_0001175 reverses HCV NS3‐Induced proliferation, migration, and EMT in liver cancer cells

**FIGURE 3:**
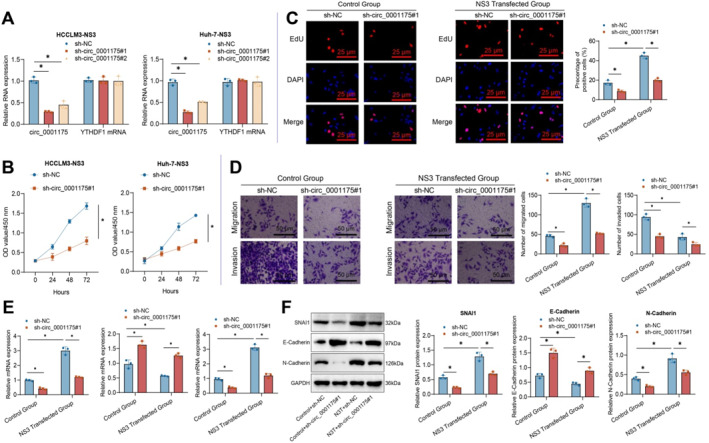
Knockdown of circ_0001175 affects the proliferation, migration, and invasion of HCC‐NS3 cells. *Note:* (A) Knockdown efficiency of sh‐circ_0001175#1 and sh‐circ_0001175#2 in HCCLM3‐NS3 and Huh‐7 cells by RT‐qPCR. (B) Cell viability of HCCLM3‐NS3 and Huh‐7 cells transfected with sh‐circ_0001175#1 or sh‐NC using the CCK‐8 assay. (C) Percentage of BrdU‐positive HCCLM3‐NS3 cells. (D) Transwell assay to measure the migration and invasion of HCCLM3‐NS3 cells. (E‐F) Expression of EMT‐related markers (SNAI1, CDH1, CDH2) analyzed by RT‐qPCR (E) and Western blot (F) following circ_0001175 knockdown. * indicates a difference between the two groups (*p* < 0.05). All cell experiments were repeated three times (*n* = 3). EMT, Epithelial‐Mesenchymal Transition; HCC, Hepatocellular carcinoma; NS3, Non‐structural protein 3; RT‐qPCR, Reverse Transcription Quantitative Polymerase Chain Reaction.

Extensive evidence indicates that HCV directly modulates oncogenic signaling pathways, leading to the development of HCC.^45^, ^46^ To explore the regulatory role of the NS3 protein in circ_0001175‐mediated effects, we investigated its impact on proliferation, migration, and invasion in HCC‐NS3 cells. RT‐qPCR results demonstrated a downregulation in the expression of circ_0001175 in HCCLM3‐NS3 and Huh‐7‐NS3 cells following transfection with shRNA circ_0001175. However, no alteration was observed in the expression of YTHDF1 mRNA (Figure 3A).

The CCK‐8 experiments demonstrated a decrease in cell proliferation following transfection with sh‐circ_0001175#1 (Figure 3B). EdU detection demonstrated a decreased percentage of EdU‐positive cells in circ_0001175‐knockdown liver cancer cells (Figure 3C; Figure S1A). Transwell assays demonstrated that the knockdown of circ_0001175 decreased the invasion and migration abilities of liver cancer cells (Figure 3D; Figure S1B). RT‐qPCR and western blot results showed that silencing circ_0001175 downregulated the expression of SNAI1 and CDH2 levels while upregulating CDH1 in HCC cells (Figure 3E–F; Figure S1C–D). Moreover, interfering with circ_0001175 reversed the promotive effects of the NS3 protein on HCC cells (Figure 3C–E; Figure S1).

These findings demonstrate that the HCV NS3 protein induces several biological phenomena, such as cell proliferation, migration, and invasion, by upregulating the expression of circ_0001175 in liver cancer cells.

### 3.4 circ_0001175 targets and binds to miR‐130a‐5p, promoting the malignant progression of HCC cells

**FIGURE 4:**
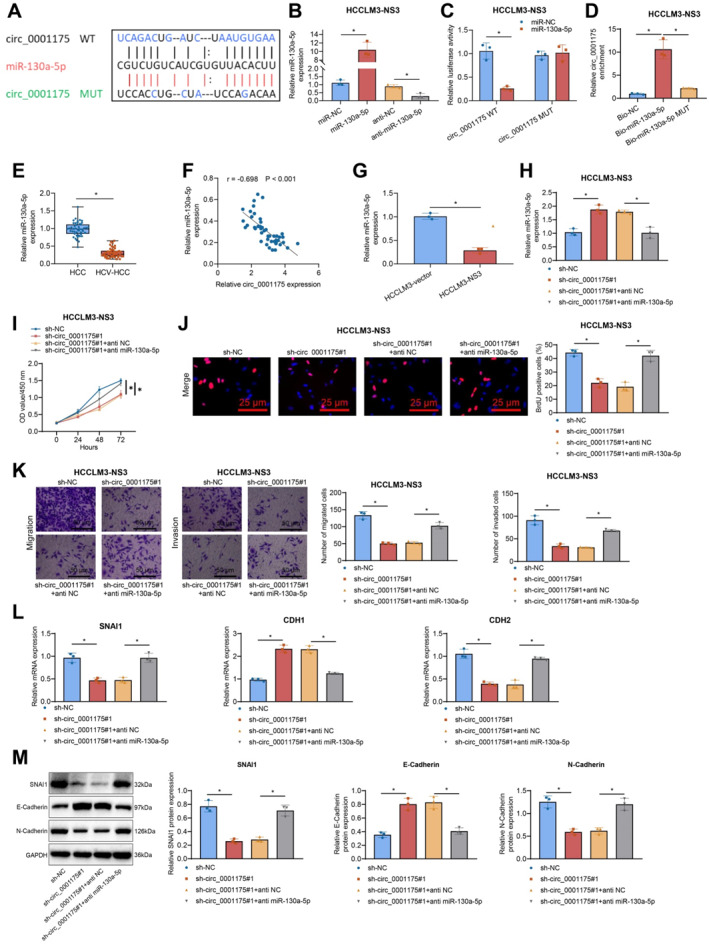
Circ_0001175 regulates the malignant progression of liver cancer cells by targeting miR‐130a‐5p. *Note:* (A) Prediction of circ_0001175 and miR‐130a‐5p binding sites through bioinformatics analysis. (B) miR‐130a‐5p expression in HCCLM3‐NS3 cells after overexpression or knockdown, detected by RT‐qPCR. (C) Validation of circ_0001175 and miR‐130a‐5p association in HCCLM3‐NS3 cells using the dual‐luciferase reporter assay. (D) Interaction between circ_0001175 and miR‐130a‐5p in HCCLM3‐NS3 cells, confirmed by RNA pull‐down assay. (E) miR‐130a‐5p expression in HCV‐negative and HCV‐positive HCC tissues, detected by RT‐qPCR (*n* = 46). (F) Correlation between circ_0001175 and miR‐130a‐5p, investigated by Pearson correlation analysis (*n* = 46). (G) miR‐130a‐5p expression in HCCLM3‐NS3 cells, measured by RT‐qPCR. (H) Expression levels of miR‐130a‐5p in cells after transfection with sh‐circ_0001175#1, sh‐circ_0001175#1 + anti‐miR‐130a‐5p, or control, detected by RT‐qPCR. (I) Cell viability of HCCLM3‐NS3 cells with different expression levels of circ_0001175 and miR‐130a‐5p, measured by CCK‐8 assay. (J) Percentage of positive cells in HCCLM3‐NS3 cells with different circ_0001175 and miR‐130a‐5p expression, assessed by EdU assay. (K) Migration and invasion of HCCLM3‐NS3 cells with different circ_0001175 and miR‐130a‐5p expression, measured by Transwell assay. (L–M) EMT‐related gene expression in HCCLM3‐NS3 cells with different circ_0001175 and miR‐130a‐5p expression, measured by RT‐qPCR and the western Blot. All cell experiments were conducted in the HCCLM3 cell line overexpressing NS3. Data are presented as mean ± SD, * indicates a comparison between the two groups of connections, with a *p*‐value of <0.05. All cell experiments were repeated three times (*n* = 3). EMT, Epithelial‐Mesenchymal Transition; NS3, Non‐structural protein 3; RNA, Ribonucleic Acid; RT‐qPCR, Reverse Transcription Quantitative Polymerase Chain Reaction.

CircRNAs are known to bind specific miRNAs, thereby alleviating the miRNA‐mediated suppression of downstream target genes.^47^ Analysis using the StarBase database predicted that circ_0001175 (also known as circYTHDF1) contains binding sites for miR‐130a‐5p. Previous studies reported that the abnormal expression of circ_0001175 and miR‐130a‐5p correlates with poor clinicopathological characteristics in HCC patients and that circ_0001175 promotes HCC progression by upregulating SNX5 expression through miR‐130a‐5p.^21^ To determine whether circ_0001175 (circYTHDF1) could interact with miR‐130a‐5p and modulate its expression in the presence of HCV NS3 transfection, we initially generated plasmids for upregulating or downregulating miR‐130a‐5p. Moreover, we constructed wild‐type and MUT plasmids encompassing the binding site between circ_0001175 and miR‐130a‐5p (Figure 4A). The experimental results from RT‐qPCR indicated an increase in the expression of miR‐130a‐5p following overexpression, whereas a decrease in the expression of miR‐130a‐5p was observed after knockdown (Figure 4B; Figure S2A). The results of the dual luciferase reporter assay indicated that the co‐transfection of wild‐type miR‐130a‐5p and circ_0001175 led to an inhibition of luciferase activity, whereas there was no change in luciferase activity upon the co‐transfection of MUT miR‐130a‐5p and circ_0001175 (Figure 4C; Figure S2B). Furthermore, Bio‐miR‐130a‐5p showed a higher binding affinity to circ_0001175 when compared to the Bio‐NC and Bio‐miR‐130a‐5p MUT groups (Figure 4D; Figure S2C).

RT‐qPCR analysis revealed lower miR‐130a‐5p expression in HCV‐positive liver cancer tissues compared to HCV‐negative tissues (Figure 4E). A negative correlation between circ_0001175 and miR‐130a‐5p expression was observed in HCV‐positive HCC patient tissues (*n* = 40) (Figure 4F). We observed a decrease in the expression of miR‐130a‐5p in liver cancer cells transfected with the NS3 protein compared to nontransfected cells (Figure 4G; Figure S2D). Furthermore, anti‐miR‐130a‐5p could reverse the increase in miR‐130a‐5p expression induced by sh‐circ_0001175#1 (Figure 4H; Figure S2E).

Functional assays indicated that transfection with anti‐miR‐130a‐5p could reverse the growth inhibitory effect caused by sh‐circ_0001175#1 in liver cancer cells (Figure 4I; Figure S2F). In comparison to the sh‐circ_0001175#1 + anti‐NC group, the sh‐circ_0001175#1 + anti‐miR‐130a‐5p group exhibited a substantial augmentation in BrdU‐positive cells (Figure 4J; Figure S2G). Furthermore, the downregulation of circ_0001175 (Figure 4K; Figure S2H) was counteracted by anti‐miR‐130a‐5p, restoring the inhibitory regulation of liver cancer cell migration and invasion. RT‐qPCR and western blot analyses demonstrate EMT‐related genes (SNAI1, CDH1, and CDH2) and were differentially expressed in the sh‐circ_0001175#1 + anti‐miR‐130a‐5p group compared to the sh‐circ_0001175#1 +anti‐NC group, confirming the regulatory role of miR‐130a‐5p (Figure 4L–M; Figure S2I–J).

In conclusion, miR‐130a‐5p is a target molecule of circ_0001175, and circ_0001175 induces the malignant progression of HCC cells by binding to and targeting miR‐130a‐5p.

### 3.5 Circ_0001175 targets miR‐130a‐5p to regulate MDM4 expression, modulating tumor progression in HCV NS3‐Positive liver cancer cells

**FIGURE 5:**
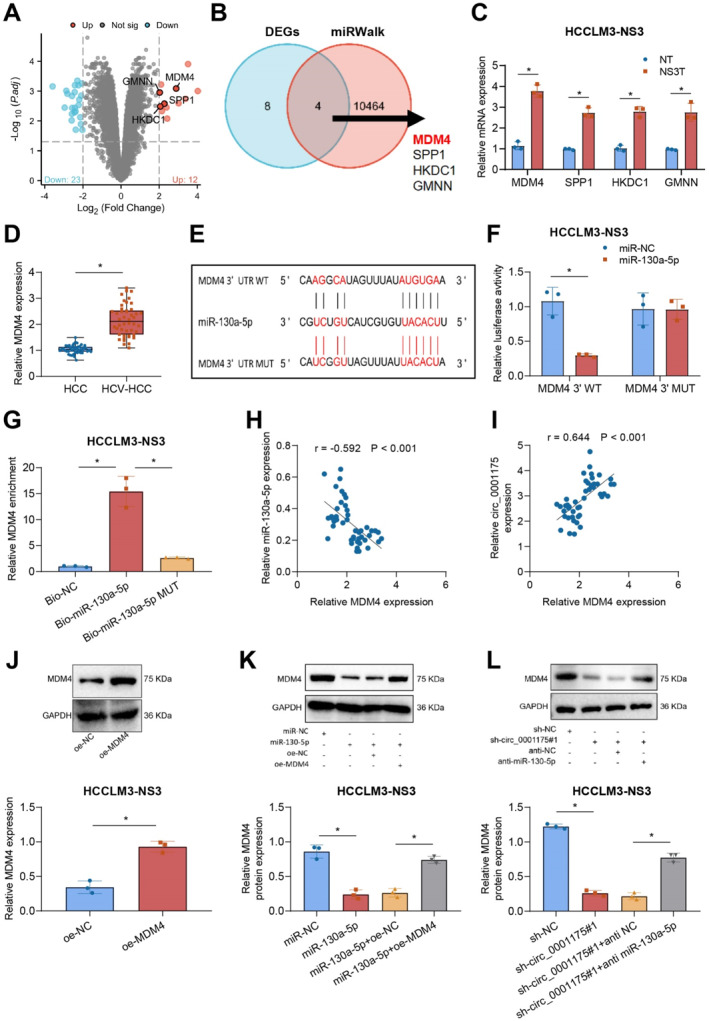
Circ_0001175 modulates the expression of MDM4 by targeting miR‐130a‐5p. *Note:* (A) Confirmation of differential gene expression in liver cancer cells transfected with NS3 through microarray analysis, revealing 12 upregulated genes and 23 downregulated genes, with the presentation of the analysis in a volcano plot. (B) Analysis of differential genes with |LogFC| > 2 and *p* < 0.05, along with miRWalk predicted target genes, using Venn diagram analysis. (C) Detection of key differential gene expression via RT‐qPCR. (D) Assessment of MDM4 expression in HCV‐negative/positive liver cancer tissues (*n* = 46) using RT‐qPCR. (E) Prediction of the binding site of miR‐130a‐5p on MDM4 mRNA by the miRBD database. (F) Investigation of the interaction between MDM4 mRNA and miR‐130a‐5p through the dual‐luciferase reporter gene assay. (G) Verification of the binding between MDM4 mRNA and miR‐130a‐5p via the RNA pull‐down experiment. (H–I) Examination of the linear relationship between MDM4 and miR‐130a‐5p/circ_0001175 using Pearson correlation analysis (*n* = 46). (J) The overexpression effect of transfected MDM4 through western blot analysis. (K) Evaluation of the impact of miR‐130a‐5p on MDM4 expression via the western blot. (L) Analysis of MDM4 protein following the transfection of sh‐circ_0001175#1, sh‐circ_0001175#1 + anti‐miR‐130a‐5p, or control group into stable NS3‐expressing liver cancer cells using the western blot. Data are presented as mean ± SD, * indicates *p* < 0.05 compared between two groups, and all cell experiments were repeated 3 times (*n* = 3). NS3, Non‐structural protein 3; RNA, Ribonucleic Acid; RT‐qPCR, Reverse Transcription Quantitative Polymerase Chain Reaction.

Research indicates that miRNAs play a pivotal role in various stages of tumor progression by downregulating target genes through degradation or translational inhibition.^48^ To further investigate the interaction between miR‐130a‐5p and circ_0001175, as well as their downstream regulatory targets, we performed differential gene expression analysis in NS3‐transfected liver cancer cells compared to nontransfected cells using transcriptome sequencing. Using the criteria of |log2FC| > 2 and *P* < 0.05, the analysis identified 12 upregulated and 23 downregulated genes in NS3‐stable liver cancer cells (Figure 5A). Target prediction through the miRWalk database, combined with Venn diagram analysis, revealed four upregulated genes—MDM4, SPP1, HKDC1, and Geminin (GMNN)—predicted to be targets of miR‐130a‐5p (Figure 5B).

Research indicates that miRNAs play a pivotal role in various stages of tumor progression by downregulating target genes by degradation or translational inhibition.^48^ To further investigate the interaction between miR‐130a‐5p and circ_0001175, as well as their downstream regulatory targets, we performed differential gene expression analysis in NS3‐transfected liver cancer cells compared to nontransfected cells using transcriptome sequencing. Using the criteria of |log2FC| > 2 and *p* < 0.05, the analysis identified 12 upregulated and 23 downregulated genes in NS3‐stable liver cancer cells (Figure 5A). The miRWalk database was used to predict targets of miR‐130a‐5p, and Venn diagram analysis was performed. The results revealed that four upregulated genes, namely MDM4, SPP1, HKDC1, and GMNN, were simultaneously predicted to be targeted by miR‐130a‐5p (Figure 5B).

Among these, MDM4 exhibited the highest expression in NS3‐transfected liver cancer cells, as confirmed by RT‐qPCR (Figure 5C; Figure S3A). Additionally, HCV‐positive liver cancer patients showed significantly elevated MDM4 mRNA levels compared to HCV‐negative patients (Figure 5D). The miRBD database further identified potential miR‐130a‐5p binding sites within MDM4 (Figure 5E). A dual‐luciferase reporter assay confirmed that miR‐130a‐5p suppressed MDM4 expression by directly binding to its 3’ UTR, as evidenced by reduced luciferase activity in MDM4 3’ UTR WT‐transfected cells but not in those transfected with a mutated plasmid (Figure 5F; Figure S3B). RNA pull‐down assays further validated the direct interaction between miR‐130a‐5p and MDM4 mRNA (Figure 5G; Figure S3C).

Pearson correlation analysis showed a negative correlation between miR‐130a‐5p and MDM4 expression, alongside a positive correlation between circ_0001175 and MDM4 expression (Figure 5H–I). Western blot analysis confirmed the upregulation of MDM4 protein levels following transfection (Figure 5J; Figure S3D) and the overexpression of MDM4 restored MDM4 protein levels that had been reduced by miR‐130a‐5p (Figure 5K; Figure S3E). Notably, silencing circ_0001175 using sh‐circ_0001175#1 decreased MDM4 protein expression, while anti‐miR‐130a‐5p partially reversed this reduction (Figure 5L; Figure S3F). Functional assays validated the role of this pathway. Overexpression of MDM4 rescued the growth inhibition caused by miR‐130a‐5p, as demonstrated by CCK‐8 assays (Figure S3G).

The miR‐130a‐5p group exhibited a decrease in BrdU‐positive cells compared to the control group (miR‐NC group). Following the overexpression of MDM4 in cells, there was an increase in the number of BrdU‐positive cells in the miR‐130a‐5p + oe MDM4 group (Figure S3H). Furthermore, the overexpression of MDM4 counteracted the inhibitory effects on migration and invasion caused by the upregulation of miR‐130a‐5p (Figure S3I–J). In contrast, the miR‐130a‐5p + oe MDM4 group reversed the mRNA and protein level alterations of EMT‐related genes in the cells compared to the miR‐130a‐5p group (Figure S3K‐N).

The above results indicate that circ_0001175 regulates the expression of MDM4 by targeting miR‐130a‐5p_3p.

### 3.6 Circ_0001175 upregulates MDM4 expression by binding to and targeting miR‐130a‐5p, thereby inhibiting the activation of the P53 pathway

The tumor suppressor protein P53, encoded by the TP53 gene, plays a crucial role as one of the body’s primary anticancer defenses. Abnormal P53 activity is common in tumors and often results from mutations or inactivation caused by MDM2/MDM4 amplification.^49^ MDM4 is a key inhibitor of P53 and acts as an oncogene when aberrantly expressed.^50^ The regulatory mechanisms of P53 and its downstream effector p21 are crucial for maintaining cellular homeostasis and suppressing abnormal cell proliferation.

**FIGURE 6:**
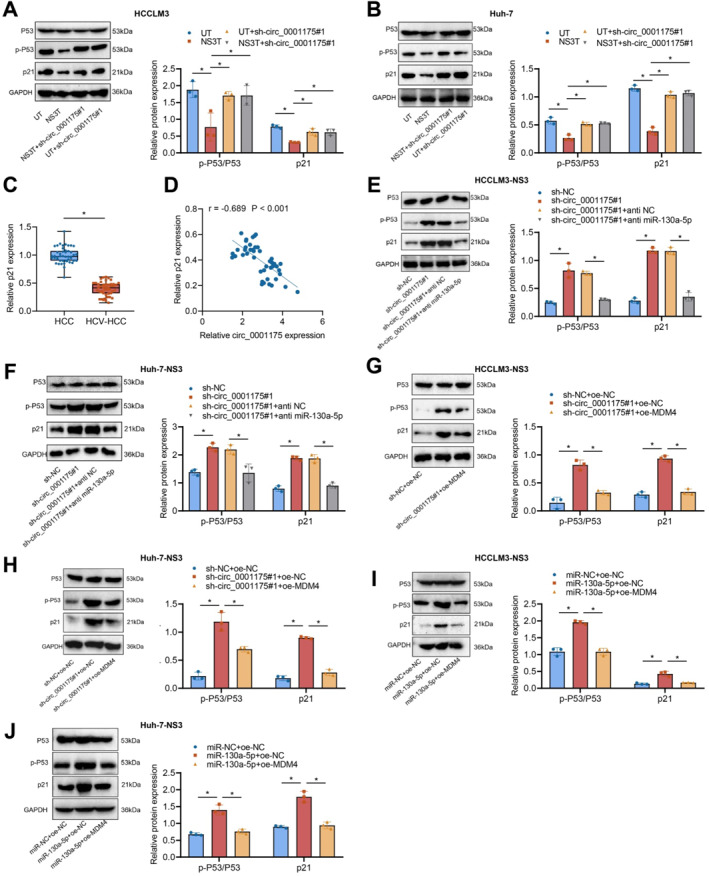
The effect of circ_0001175 on activating the P53 pathway via the miR‐130a‐5p/MDM4 axis. *Note:* (A–B) Expression of p‐P53, P53, and p21 via the western blot. (C) Differential expression of p21 in HCV‐positive and HCV‐negative liver cancer tissues (*n* = 40) using RT‐qPCR. (D) The linear relationship between p21 and circ_0001175 through Pearson correlation analysis (*n* = 40). (E–F) The impact of sh‐circ‐0001175#1, sh‐circ‐0001175#1+anti‐miR‐130a‐5p, and control groups on P53 phosphorylation and p21 expression levels using the western blot. (G–H) The effect of sh‐circ‐0001175#1, sh‐circ‐0001175#1+oe‐MDM4, and control groups on P53 phosphorylation and p21 expression levels through the western blot. (I–J) Expression levels of p‐P53, P53, and p21 via the western blot in miR‐130a‐5p + oe‐NC, miR‐130a‐5p + oe‐MDM4, and control groups. *p < 0.05 indicates significance when comparing two groups. All cell experiments were repeated three times (*n* = 3); data are presented as mean ± SD. RT‐qPCR, Reverse Transcription Quantitative Polymerase Chain Reaction.

To examine the roles of NS3 and circ_0001175 in the P53 pathway, we observed significant reductions in P53 activity and p21 levels in NS3‐overexpressing liver cancer cells. Knocking down circ_0001175 restored P53 activity, indicating that circ_0001175 inhibits P53 activation (Figure 6A–B). The RT‐qPCR analysis of clinical samples showed reduced p21 expression in HCV‐positive liver cancer tissues compared to HCV‐negative tissues (Figure 6C). A negative correlation between circ_0001175 and p21 expression was also observed (Figure 6D). Dual knockdown of circ_0001175 and miR‐130a‐5p suppressed P53 activity, confirming that circ_0001175 binds to and inhibits miR‐130a‐5p to suppress P53 activation (Figure 6E–F). Overexpression of MDM4 in circ_0001175‐knockdown cells further reduced P53 activity, highlighting MDM4’s role in P53 pathway regulation (Figure 6G–H). Under conditions of miR‐130a‐5p overexpression and MDM4 inhibition, P53 phosphorylation was partially restored, along with increased p21 expression (Figure 6I–J).

In conclusion, our experiment showed that HCV NS3 is involved in the functioning of HCC through the circ_0001175/miR‐130a‐5p/MDM4/P53 pathway. It offers a fresh perspective on the mechanisms through which HCV NS3 promotes the progression of HCC.

### 3.7 In Vivo confirmation of HCV NS3‐mediated HCC progression through the circ_0001175/miR‐130a‐5p/MDM4/P53 axis and its impact on tumor metastasis

**FIGURE 7:**
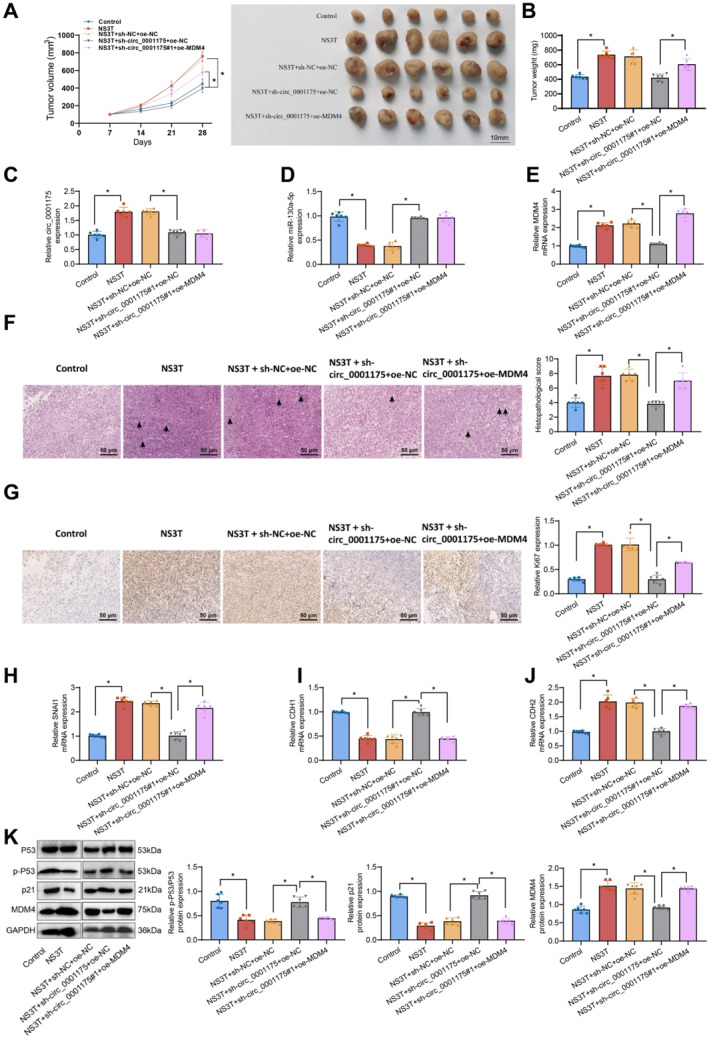
The influence of NS3 on tumor growth and metastasis via the circ_0001175/miR‐130a‐5p/MDM4/P53 axis in vivo. *Note:* (A–B) Tumor volume and weight after knocking out circ_0001175 in (A–B), knocking out circ_0001175, and overexpressing MDM4. (C–E) Relative expression levels of circ_0001175, miR‐130a‐5p, and MDM4 in tumor tissues of each group were detected by RT‐qPCR. (F) Histopathological sections of tumor and normal tissues with pathological scoring, black arrows indicating areas of nuclear condensation, rupture, or fragmentation. (G) Immunohistochemical analysis of the Ki67 positivity rate with semiquantitative analysis. (H–J) Relative expression levels of SNAI1, CDH1, and CDH2 in tumor tissues of each group were detected by RT‐qPCR. (K) Protein levels of MDM4, P53, p‐P53, and p21 in tumor tissues were analyzed by the western blot. Data are presented as mean ± SD, * indicates *p* < 0.05 compared between the two groups, with 6 mice in each group (*n* = 6). NS3, Non‐structural protein 3; RT‐qPCR, Reverse Transcription Quantitative Polymerase Chain Reaction.

To confirm the regulatory role of the circ_0001175/miR‐130a‐5p/MDM4 axis in NS3‐mediated liver cancer progression, we established a xenograft model. The tumor growth rate and size were significantly increased in the NS3T group compared to controls, whereas circ_0001175 knockdown inhibited tumor growth. Overexpression of MDM4 partially rescued this effect (Figure 7A–B). RT‐qPCR analysis confirmed increased circ_0001175 and MDM4 expression and reduced miR‐130a‐5p expression in NS3T tumors. Circ_0001175 knockdown increased miR‐130a‐5p expression and decreased MDM4 levels, whereas MDM4 overexpression restored MDM4 protein expression without affecting circ_0001175 or miR‐130a‐5p levels (Figure 7C–E). Histological and immunohistochemical analyses revealed increased Ki67 expression and nuclear abnormalities in NS3T tumor tissues, which were reversed by circ_0001175 knockdown. However, MDM4 overexpression abolished these changes (Figure 7F–G). RT‐qPCR analysis demonstrated a significant downregulation of SNAI1 and CDH2 expression and an upregulation of CDH1 expression, following circ_0001175 knockdown. Overexpression of MDM4 partially restored these changes (Figure 7H–J). Western blot analysis confirmed reduced MDM4 and increased p‐P53 and p21 levels after circ_0001175 knockdown, which were reversed by MDM4 overexpression (Figure 7K). These findings suggest that circ‐0001175 plays a crucial role as a mediator in HCC and NS3. Overexpression of MDM4 partially counteracts the effects of circ_0001175 knockdown, further confirming in vivo that circ_0001175 upregulates MDM4 expression by targeting and binding to miR‐130a‐5p.

Finally, in a lung metastasis model, NS3T cells significantly increased lung metastases compared to controls. Knockdown of circ_0001175 reduced metastasis, whereas MDM4 overexpression exacerbated it (Figure S4A‐C).

In summary, our study uncovered a novel regulatory network in which the HCV NS3 protein stimulates the expression of circ‐0001175. It leads to regulating the P53 pathway via the miR‐130a‐5p/MDM4 axis, subsequently promoting the progression of HCC.

## 4 DISCUSSION

In recent years, increasing attention has been focused on the association between HCC and HCV infection.^51^, ^52^, ^53^ Of particular interest is the role of the HCV NS3 protein in this interaction. Several studies have highlighted its significant involvement in the virus’s processing and maturation, making it a promising target for antitumor therapy.^54^ However, there has been ongoing debate regarding how the HCV NS3 protein interferes with the molecular mechanisms of liver cancer. This study addresses this controversy by providing crucial insights into the molecular mechanisms through which the HCV NS3 protein participates in the growth and metastasis of liver cancer by regulating the circ_0001175/miR‐130a‐5p/MDM4/P53 axis, thereby contributing to the enrichment and enhancement of research in this field.^20^

Although previous studies have demonstrated a strong association between HCV and liver cancer,^52^, ^53^, ^55^ much of this work has mainly focused on isolated pathways or molecules, such as the cleavage of MAVS^56^ or the degradation of TRIF,^57^ or focused on immune escape mechanisms.^58^ These studies have lacked a comprehensive and systematic analytical framework.^59^ In comparison to previous studies, our research uniquely proposes and validates, for the first time, that the HCV NS3 protein influences the growth and metastasis of liver cancer by regulating the circ_0001175/miR‐130a‐5p/MDM4/P53 axis.

This study identified a correlation between HCV NS3 protein and the expression of circ_0001175. Subsequent cellular experiments validated the role of NS3 protein in augmenting the proliferation, migration, and invasion capabilities of liver cancer cells through the induction of circ_0001175 expression. This mechanism elucidates the increased susceptibility of HCV‐infected individuals to the development of liver cancer, offering novel targets for the prospective treatment of HCV‐related liver cancer. Although we observed significantly upregulated circ_0001175 expression in HCCLM3‐NS3 and HCCLM3‐vector liver cancer cell samples, the specific mechanism by which HCV NS3 upregulates circ_0001175 expression requires further verification.

Through the use of whole transcriptome sequencing technology and analysis of liver cancer cells, this study successfully identified potential key genes associated with the occurrence and progression of HCV‐related HCC. The advantage of this method lies in its ability to comprehensively capture information from the entire transcriptome, greatly enhancing the accuracy and comprehensiveness compared to traditional targeted sequencing technologies.

The study by Siavoshian S et al. demonstrated that HCV NS3 protein exhibits antiproliferative properties by inducing cell cycle arrest.^60^ Their research employed a transient overexpression model mediated by recombinant adenovirus, whereas our study utilized stable transfection and lentiviral infection models in HCCLM3 and Huh‐7 cell lines. These differences may account for the varying functional effects of NS3. For instance, transient overexpression might activate stress signals leading to cell cycle arrest, whereas in our models, the physiological levels of NS3 better mimic chronic HCV infection, promoting oncogenic pathways. Furthermore, although Siavoshian S et al. reported that NS3’s antiproliferative effect is p53‐independent, our findings reveal that NS3 downregulates p53 and p21 expression via the circ_0001175/miR‐130a‐5p/MDM4 pathway, thereby facilitating HCC cell proliferation and metastasis. These discrepancies may be attributed to the genetic background of the cell lines or the functional diversity of NS3 in different cellular contexts. Beyond its regulation of circRNAs, NS3 protein also contributes to tumorigenesis through other mechanisms. For example, the NS3/4A protease persistently activates EGFR signaling by inhibiting T‐cell protein tyrosine phosphatase, thereby promoting invasive development post‐HCV infection.^31^ HCV proteins (Core, NS3, NS4, and NS5A) are extensively involved in processes such as cell signaling, DNA repair, transcription and translation regulation, proliferation, and apoptosis. Core and NS5A, in particular, activate the *β*‐catenin pathway, a key target in viral‐induced transformation.^61^ Additionally, NS3 promotes HCC cell migration and invasion by inducing ubiquitination and degradation of PPM1A, representing a novel carcinogenic strategy of HCV.^46^ While this study primarily focuses on the regulation of circ_0001175 by NS3 and its downstream pathways, future research should investigate the broader roles of NS3 in other signaling pathways, including both p53‐dependent and independent mechanisms. The expression levels and duration of NS3 under different experimental conditions may also significantly influence its functional effects, which warrants further exploration.

**FIGURE 8:**
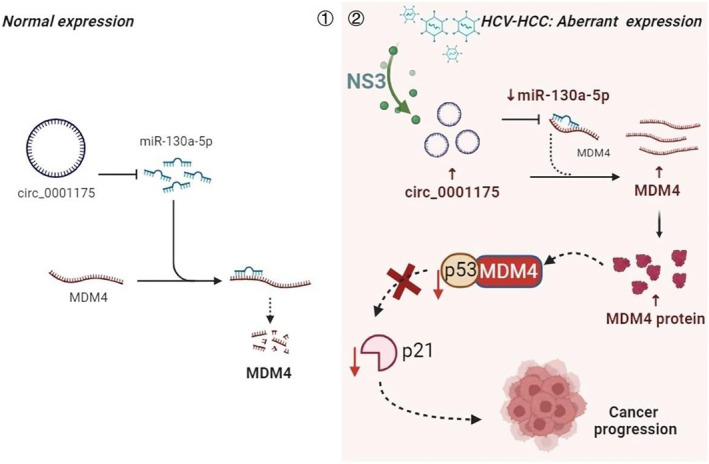
The hepatitis C virus NS3‐mediated circ_0001175/miR‐130a‐5p/MDM4/P53 axis promotes HCV‐HCC development. *Note:* (1) The expression of circ_0001175 is lower in tissues without NS3 transfection. MiR‐130a‐5p could bind to MDM4 mRNA, thereby inhibiting translation and decreasing MDM4 protein levels. (2) NS3 increases circ_0001175 levels, which further act as a “sponge” for miR‐130a‐5p, reducing its levels. Consequently, miR‐130a‐5p can no longer target MDM4 mRNA, leading to increased MDM4 translation. This inhibition of P53 activity results in the suppression of p21 transcription, leading to decreased p21 expression and the promotion of uncontrolled cell proliferation, ultimately contributing to HCC progression. HCC, Hepatocellular carcinoma; NS3, Non‐structural protein 3.

This study systematically reveals the central role played by HCV NS3 protein in the growth and metastasis of HCC, particularly through the circ_0001175/miR‐130a‐5p/MDM4/P53 axis. We found that HCV NS3 protein significantly enhances circ_0001175 expression in HCCLM3, Huh‐7, and liver cancer tissues, which, in turn, targets and binds to miR‐130a‐5p. This led to the upregulation of MDM4 and inhibition of the P53 pathway at both transcriptional and protein levels, affecting downstream pathways by significantly downregulating SNAI1 and CDH2 expression while upregulating CDH1. Additionally, Ki67 expression increased, which confirmed *in both* in vitro and in vivo experiments that NS3 promotes liver cancer growth and metastasis (Figure 8). Using the miRWalk database for miR‐130a‐5p target prediction and Venn diagram analysis, we identified four upregulated genes in the differential gene set—MDM4, SPP1, HKDC1, and GMNN—as potential targets of miR‐130a‐5p (Figure 5). Therefore, we speculate that in addition to the circ_0001175/miR‐130a‐5p/MDM4/P53 axis, circ_0001175/miR‐130a‐5p may also regulate liver cancer development through SPP1, HKDC1, and GMNN, though further experimental validation is required.

This study reveals the molecular mechanism by which the HCV NS3 protein promotes HCC growth and metastasis by regulating the circ_0001175/miR‐130a‐5p/MDM4/P53 pathway. These findings provide a deeper understanding of how HCV infection affects HCC progression at the cellular and molecular levels. The discovery of the interaction between the HCV NS3 protein and circ_0001175, as well as its regulation of the miR‐130a‐5p/MDM4/P53 axis, introduces a novel molecular mechanism. This insight offers new opportunities for targeted therapies against HCC, as future therapeutic strategies could focus on designing drugs or treatments targeting these key molecules. For patients with HCV infection, early screening based on the molecular pathways identified in this study could facilitate the early diagnosis and treatment of liver cancer, ultimately improving survival rates.

Although this study’s conclusions are robustly supported by experiments in human liver cancer cell lines (HCCLM3 and Huh‐7) and subcutaneous xenograft models, these models cannot fully replicate the complexity of the human physiological environment. Future research will require the validation of these findings in larger clinical samples and models that better simulate human conditions. Additionally, although this study focuses on the effects of HCV NS3 protein on HCC progression through the circ_0001175/miR‐130a‐5p/MDM4/P53 axis, other molecular pathways likely contribute to HCC progression in vivo. Further studies exploring the interactions between circ_0001175 and other pathways are necessary to achieve a more comprehensive understanding of the complex mechanisms driving liver cancer.

This study is significant in uncovering a novel role of HCV NS3 protein in HCC progression and providing insights into how it influences liver cancer through specific molecular pathways. These findings not only open new avenues for molecular biology research on HCC but also offer potential therapeutic strategies, particularly for HCV‐related liver cancer. Based on the findings of this study, future efforts could focus on drug screening and development targeting the circ_0001175/miR‐130a‐5p/MDM4/P53 pathway while exploring the potential applications of these molecules in early diagnosis, treatment, and prognosis evaluation. Although there is still a gap between laboratory research and clinical applications, we believe that further validation of these molecules in human studies will provide new possibilities for treating HCV‐related HCC.

## AUTHOR CONTRIBUTIONS

Jun Zhang and Jian Luo designed and supervised the study. Liya Hu performed the experiments, including transcriptome sequencing, qRT‐PCR, and western blot analyses. Jun Zhang and Liya Hu conducted data analysis and interpretation. Yajun Liang contributed to clinical data collection and provided critical insights into the study design. All authors contributed to manuscript drafting, revision, and approved the final version for submission.

## CONSENT TO PARTICIPATE

Not applicable.

## CONFLICT OF INTEREST STATEMENT

The author declares no conflict of interest.

## ETHICS STATEMENT

This study has received approval from the Clinical Ethics Committee of our institute (TJ‐IRB20230929) and has obtained informed consent from the patients in strict adherence to the requirements of the Helsinki Declaration. The experimental procedure and animal usage plan have been approved by the Animal Ethics Committee (TJ‐IRB20230929).

## Supporting information

Supplementary Material

## Data Availability

All data can be provided as needed.
