# Supplementary material for: NS3 of hepatitis C virus drives hepatocellular carcinoma progression through a novel RNA‐interference pathway
Source: J Cell Commun Signal. 2025 Apr 12;19(2):e70013. doi: 10.1002/ccs3.70013 (PMC11993122; doi:10.1002/ccs3.70013)
Supplement: Supplementary file 1 — Supplementary Material [file CCS3-19-e70013-s001.docx]

**
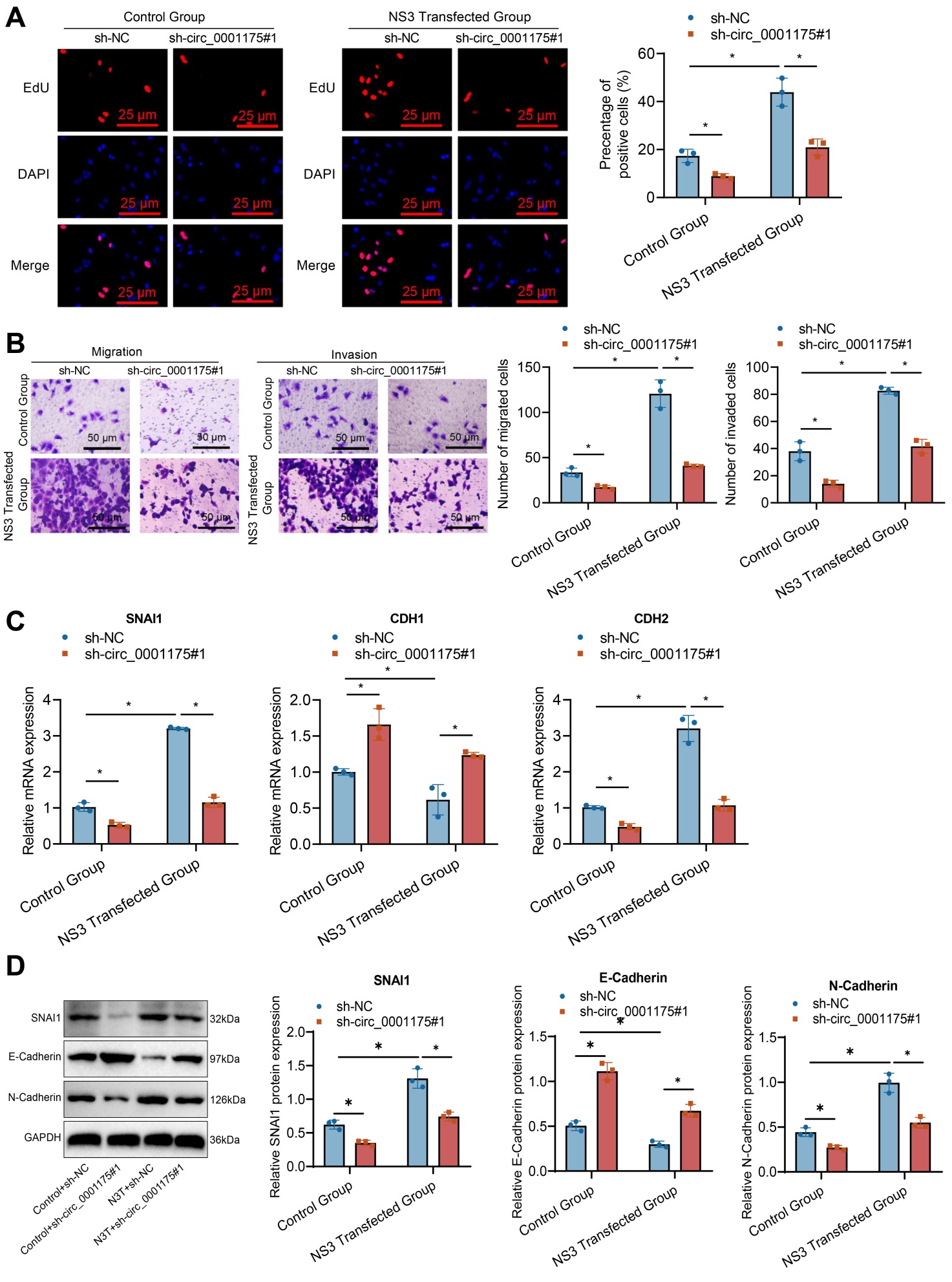
**

**Figure S1. Knockdown of circ_0001175 affects proliferation, migration, and invasion of Huh-7-NS3 cells.**

Note: (A) EdU assay to determine the percentage of Huh-7-NS3 positive cells. (B) Transwell assay to measure migration and invasion of Huh-7-NS3 cells. (C-D) RT-qPCR and Western Blot analysis of mRNA levels of EMT-related genes in Huh-7-NS3 cells. * indicates *P* < 0.05 compared between the two groups. All cell experiments were repeated three times (n = 3).

**
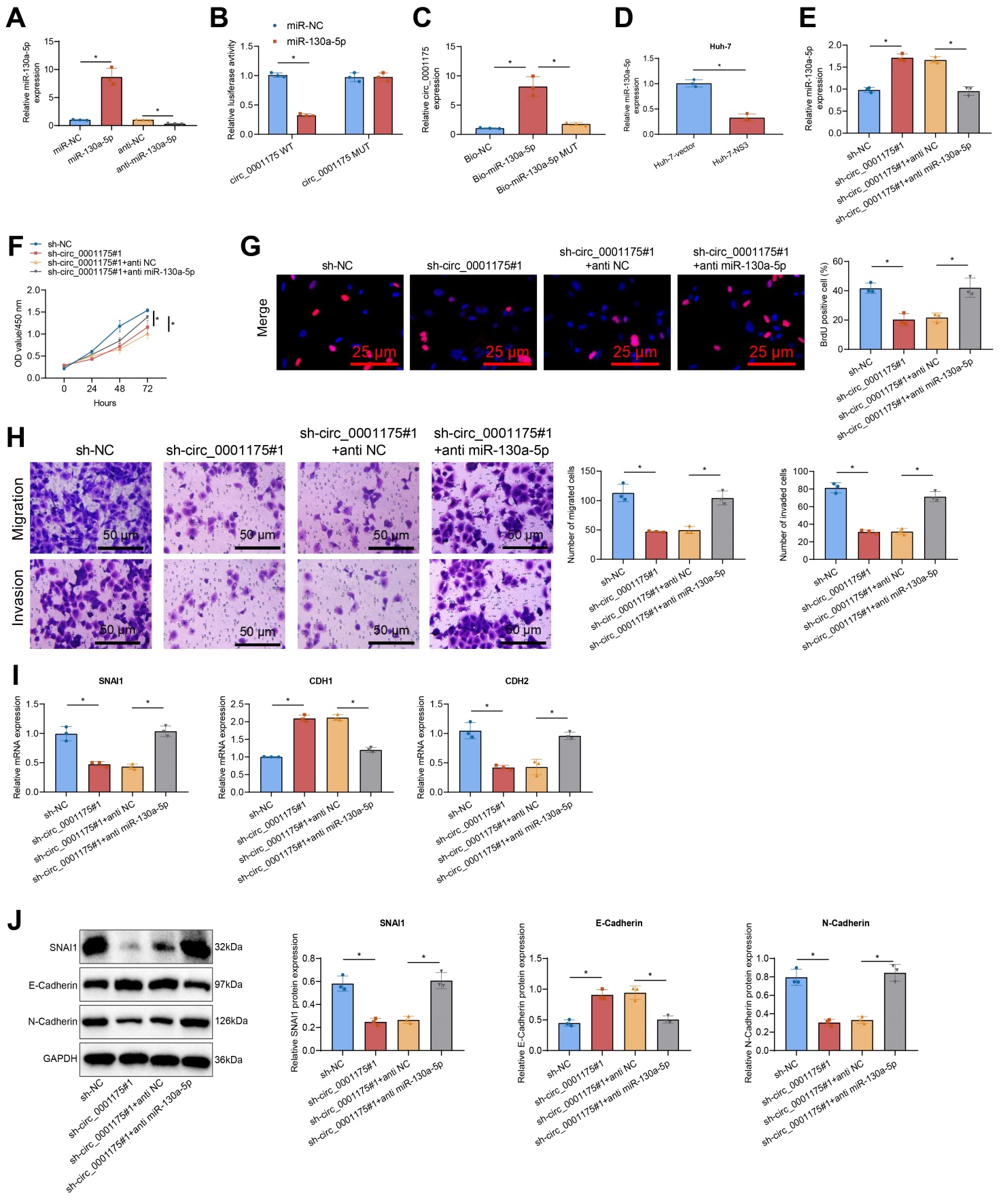
**

**Figure S2. Circ_0001175 regulates malignant progression in Huh-7 cells by targeting miR-130a-5p.**

Note: (A) The expression levels of miR-130a-5p in Huh-7 cells were detected by RT-qPCR following overexpression or knockdown; (B) Dual-luciferase reporter gene assay confirmed an association between circ_0001175 and miR-130a-5p in Huh-7 cells; (C) RNA pulldown assay in Huh-7 cells detected the interaction between circ_0001175 and miR-130a-5p; (D) RT-qPCR was used to detect the expression of miR-130a-5p in Huh-7 cells; (E) RT-qPCR was used to quantify miR-130a-5p after transfection with sh-circ_0001175#1, sh-circ_0001175#1 + anti-miR-130a-5p, or respective controls; (F) Cell viability was assessed using the CCK-8 assay; (G) The percentage of EdU-positive cells was determined using the EdU assay; (H) Cell migration and invasion were evaluated through the Transwell assay; (I-J) RT-qPCR and Western Blot was used to analyze the expression of EMT-related genes; all cell experiments were conducted in Huh-7 cell lines overexpressing NS3; data are presented as mean ± SD, * indicates *P* < 0.05 compared between two groups, and all cell experiments were repeated three times (n = 3).

**
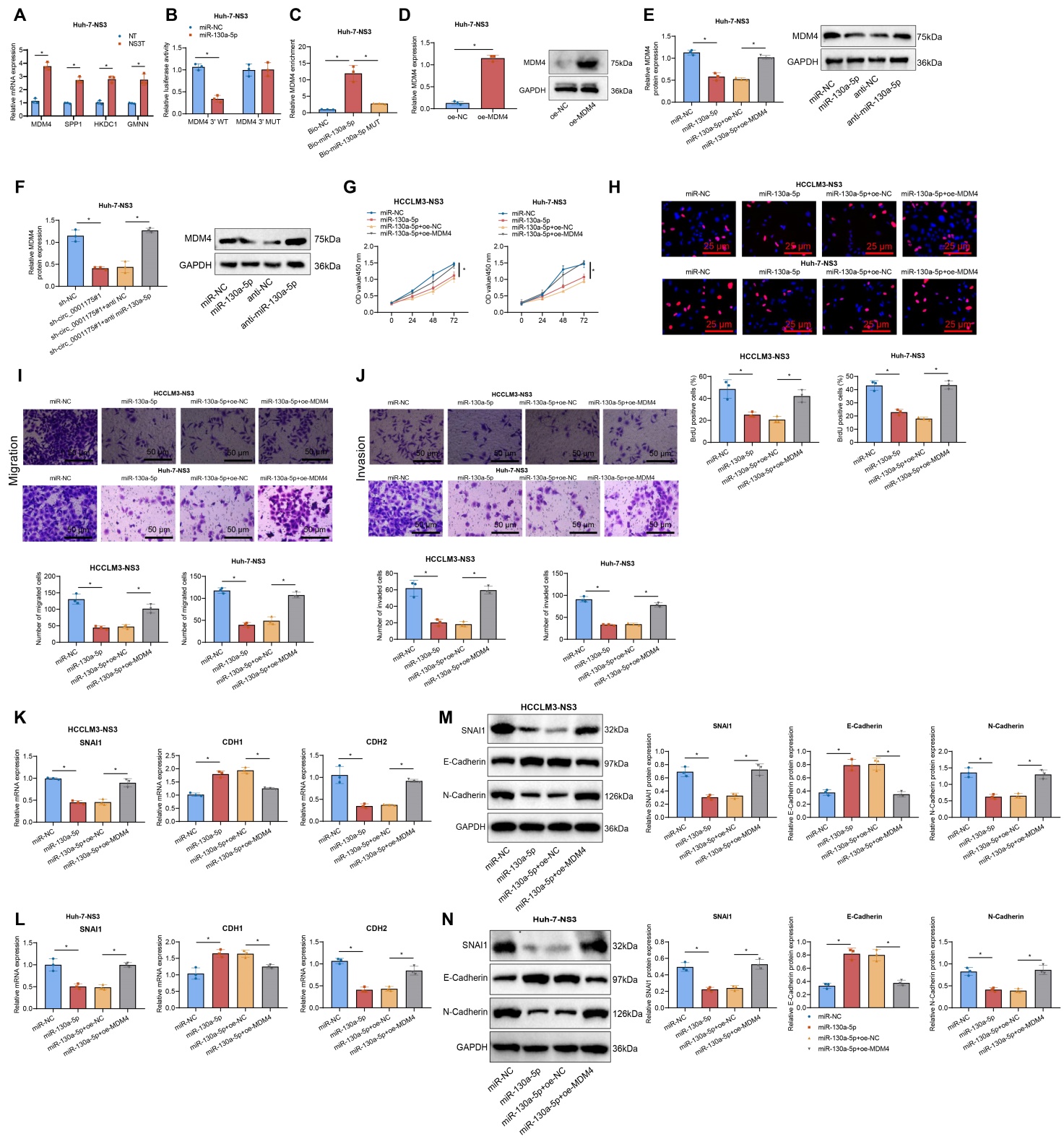
**

**Figure S3. Circ_0001175 regulates MDM4 expression through targeting miR-130a-5p.**

Note: (A) RT-qPCR analysis of expression of key differentially expressed genes. (B) Dual-luciferase reporter gene assay confirming the interaction between MDM4 mRNA and miR-130a-5p in Huh-7 cells. (C) RNA pulldown assay confirming the binding of MDM4 mRNA and miR-130a-5p in Huh-7 cells. (D) Western blot analysis to detect MDM4 overexpression in transfected Huh-7 cells. (E) Western blot analysis to assess the impact of miR-130a-5p on MDM4 expression in Huh-7 cells. (F) Western blot analysis of MDM4 protein after transfection with sh-circ_0001175#1, sh-circ_0001175#1 + anti-miR-130a-5p, or control in stable NS3 overexpressing Huh-7 cells. (G) CCK-8 assay to measure cell viability. (H) EdU assay to determine the percentage of positive cells. (I-J) Transwell assay to evaluate cell migration and invasion. (K-L) RT-qPCR analysis of gene expression related to EMT. (M-N) Western Blot analysis of gene expression related to EMT. Data is presented as mean ± SD; * indicates *P* < 0.05 compared between the two groups. All cell experiments were repeated three times (n = 3).

**
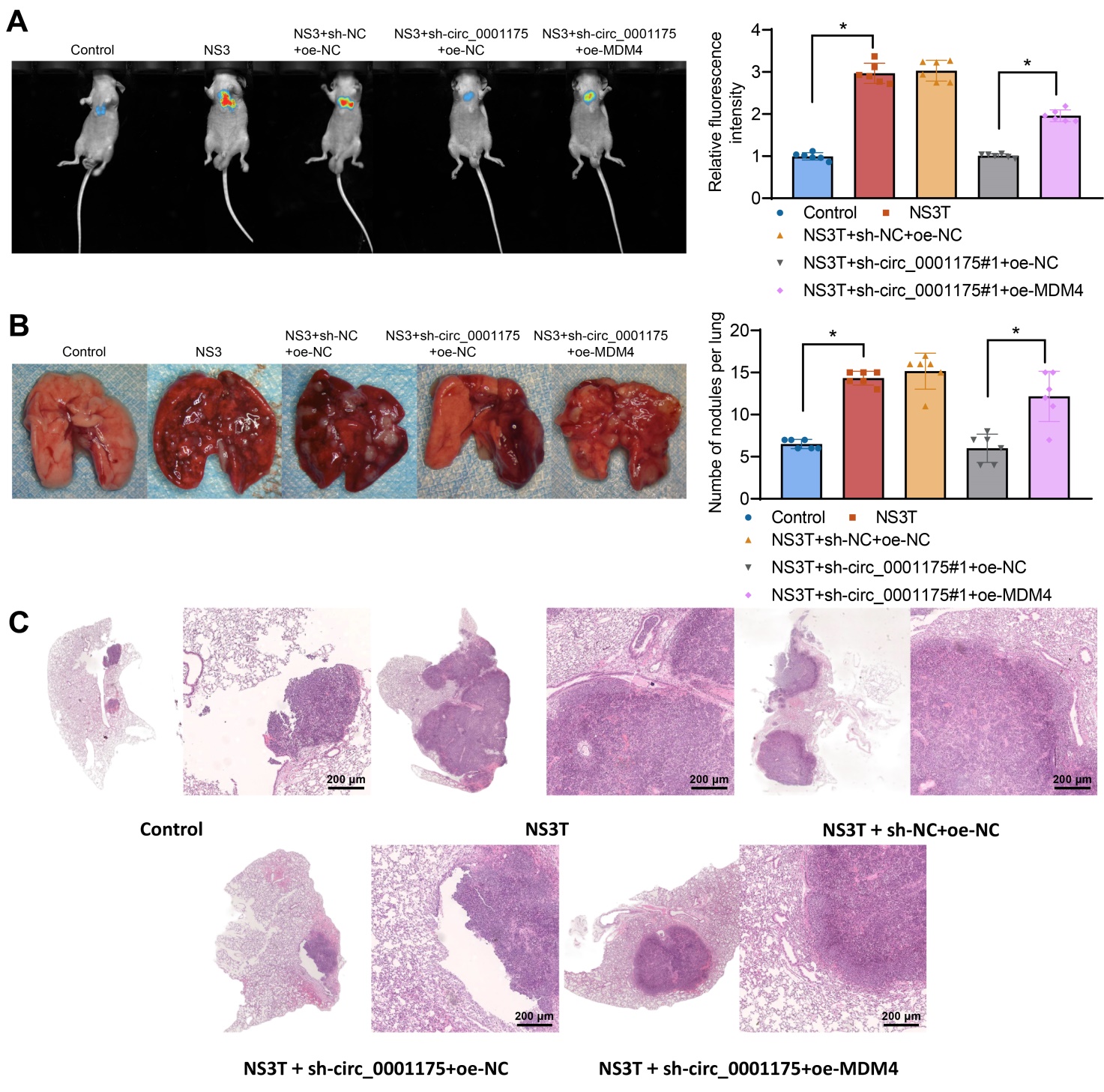
**

**Figure S4. NS3 affects tumor growth and metastasis *in vivo* through the circ_0001175/miR-130a-5p/MDM4/P53 axis.**

Note: (A) *In vivo* bioluminescence imaging of mouse tumor growth. (B) Several pulmonary metastatic nodules. (C) H&E staining images of lungs extracted from mice. * indicates *P* < 0.05. Each group consisted of six mice (n = 6); data are presented as mean ± SD.

**Table S1. Patient details information**

| Patient ID | Admission Date | Years with HCV Infection | Tumor Stage | Metastasis | Pathological Markers |
| --- | --- | --- | --- | --- | --- |
| P1 | 2023−05−15 | 4 | Stage II | No | AFP Normal |
| P2 | 2022−12−29 | 1 | Stage I | No | AFP Normal |
| P3 | 2022−12−27 | 9 | Stage IV | Yes | AFP High |
| P4 | 2022−09−03 | 2 | Stage II | Yes | AFP Normal |
| P5 | 2022−12−20 | 3 | Stage II | No | AFP High |
| P6 | 2023−04−25 | 9 | Stage IV | Yes | AFP High |
| P7 | 2022−12−21 | 2 | Stage II | No | AFP Normal |
| P8 | 2022−11−26 | 1 | Stage I | No | AFP Normal |
| P9 | 2023−06−13 | 4 | Stage II | No | AFP High |
| P10 | 2023−04−20 | 2 | Stage I | No | AFP Normal |
| P11 | 2023−06−08 | 6 | Stage IV | No | AFP High |
| P12 | 2023−04−5 | 6 | Stage III | No | AFP High |
| P13 | 2023−08−17 | 8 | Stage III | Yes | AFP Normal |
| P14 | 2023−02−28 | 3 | Stage I | No | AFP Normal |
| P15 | 2023−07−29 | 1 | Stage I | Yes | AFP Normal |
| P16 | 2023−03−10 | 2 | Stage II | Yes | AFP Normal |
| P17 | 2023−03−11 | 8 | Stage III | Yes | AFP High |
| P18 | 2023−06−14 | 6 | Stage IV | No | AFP High |
| P19 | 2023−05−17 | 7 | Stage III | Yes | AFP High |
| P20 | 2023−07−20 | 10 | Stage IV | Yes | AFP High |
| P21 | 2023−02−06 | 8 | Stage III | Yes | AFP High |
| P22 | 2022−10−13 | 6 | Stage III | No | AFP High |
| P23 | 2023−07−12 | 7 | Stage IV | Yes | AFP Normal |
| P24 | 2022−09−22 | 8 | Stage IV | Yes | AFP High |
| P25 | 2023−01−04 | 8 | Stage IV | No | AFP High |
| P26 | 2023−05−16 | 1 | Stage I | No | AFP Normal |
| P27 | 2022−12−03 | 1 | Stage I | No | AFP High |
| P28 | 2023−06−10 | 2 | Stage II | No | AFP High |
| P29 | 2022−08−25 | 3 | Stage II | No | AFP Normal |
| P30 | 2023−03−14 | 5 | Stage II | No | AFP Normal |
| P31 | 2023−05−06 | 7 | Stage III | No | AFP High |
| P32 | 2023−08−08 | 9 | Stage III | Yes | AFP High |
| P33 | 2023−09−05 | 1 | Stage I | No | AFP Normal |
| P34 | 2023−07−18 | 1 | Stage I | Yes | AFP Normal |
| P35 | 2023−03−15 | 1 | Stage I | No | AFP Normal |
| P36 | 2023−09−04 | 2 | Stage II | No | AFP High |
| P37 | 2023−02−22 | 4 | Stage II | No | AFP Normal |
| P38 | 2022−09−21 | 3 | Stage II | Yes | AFP Normal |
| P39 | 2023−06−06 | 8 | Stage III | No | AFP Normal |
| P40 | 2023−07−08 | 8 | Stage III | Yes | AFP Normal |
| P41 | 2023−07−21 | 2 | Stage II | No | AFP Normal |
| P42 | 2023−07−30 | 9 | Stage IV | Yes | AFP Normal |
| P43 | 2022−11−24 | 6 | Stage III | Yes | AFP High |
| P44 | 2023−03−01 | 8 | Stage III | Yes | AFP High |
| P45 | 2022−11−26 | 8 | Stage IV | Yes | AFP High |
| P46 | 2023−03−06 | 6 | Stage III | Yes | AFP High |
| P47 | 2023−06−18 | N/A | Stage III | Yes | AFP High |
| P48 | 2023−08−16 | N/A | Stage II | No | AFP Normal |
| P49 | 2023−06−12 | N/A | Stage I | No | AFP Normal |
| P50 | 2023−07−17 | N/A | Stage II | Yes | AFP Normal |
| P51 | 2022−09−25 | N/A | Stage IV | No | AFP High |
| P52 | 2023−06−28 | N/A | Stage II | No | AFP Normal |
| P53 | 2022−11−22 | N/A | Stage II | No | AFP Normal |
| P54 | 2022−09−27 | N/A | Stage I | No | AFP Normal |
| P55 | 2023−05−09 | N/A | Stage IV | Yes | AFP High |
| P56 | 2022−11−03 | N/A | Stage IV | Yes | AFP High |
| P57 | 2022−10−14 | N/A | Stage I | No | AFP Normal |
| P58 | 2022−10−18 | N/A | Stage III | Yes | AFP High |
| P59 | 2023−08−06 | N/A | Stage IV | Yes | AFP High |
| P60 | 2023−05−14 | N/A | Stage I | No | AFP Normal |
| P61 | 2023−05−5 | N/A | Stage II | Yes | AFP Normal |
| P62 | 2023−08−10 | N/A | Stage IV | Yes | AFP High |
| P63 | 2022−12−26 | N/A | Stage II | Yes | AFP High |
| P64 | 2023−07−15 | N/A | Stage II | No | AFP Normal |
| P65 | 2023−03−26 | N/A | Stage I | No | AFP High |
| P66 | 2022−11−27 | N/A | Stage III | Yes | AFP Normal |
| P67 | 2023−09−07 | N/A | Stage II | Yes | AFP High |
| P68 | 2022−10−21 | N/A | Stage I | Yes | AFP Normal |
| P69 | 2023−03−14 | N/A | Stage I | No | AFP Normal |
| P70 | 2022−10−24 | N/A | Stage II | Yes | AFP Normal |
| P71 | 2023−05−18 | N/A | Stage I | No | AFP Normal |
| P72 | 2023−04−02 | N/A | Stage II | No | AFP Normal |
| P73 | 2023−03−02 | N/A | Stage III | Yes | AFP High |
| P74 | 2023−01−16 | N/A | Stage I | No | AFP Normal |
| P75 | 2023−03−16 | N/A | Stage III | Yes | AFP High |
| P76 | 2023−07−11 | N/A | Stage I | No | AFP Normal |
| P77 | 2022−12−30 | N/A | Stage III | Yes | AFP High |
| P78 | 2023−08−14 | N/A | Stage I | No | AFP Normal |
| P79 | 2023−04−01 | N/A | Stage I | No | AFP High |
| P80 | 2022−11−14 | N/A | Stage IV | No | AFP High |
| P81 | 2022−09−10 | N/A | Stage IV | Yes | AFP High |
| P82 | 2023−01−05 | N/A | Stage I | No | AFP Normal |
| P83 | 2023−01−06 | N/A | Stage III | No | AFP Normal |
| P84 | 2023−05−15 | N/A | Stage III | Yes | AFP High |
| P85 | 2023−01−10 | N/A | Stage I | No | AFP Normal |
| P86 | 2022−08−27 | N/A | Stage I | No | AFP Normal |
| P87 | 2022−09−20 | N/A | Stage I | No | AFP Normal |
| P88 | 2023−01−15 | N/A | Stage I | No | AFP Normal |
| P89 | 2023−05−19 | N/A | Stage I | No | AFP Normal |
| P90 | 2022−08−28 | N/A | Stage I | No | AFP Normal |
| P91 | 2023−01−22 | N/A | Stage I | No | AFP Normal |
| P92 | 2023−08−18 | N/A | Stage I | No | AFP Normal |

**Table S2. shRNA interference sequence**

| Name | sh-RNA Sequences（5’-3’） |
| --- | --- |
| sh-circ_0001175#1 | GCAGAAATTGTGTTCTTAAAT |
| sh-circ_0001175#2 | ATTCTGTGAGACTTCATATTT |

**Table S3. qRT-PCR primer sequence**

| Gene | primer sequence |
| --- | --- |
| circ_0001175（Human） | Forward: 5’- CACGTGCCCTTGTAGAGGAT -3’ |
|  | Reverse: 5’- GTCAGAGGGTCTTTTGGAGCA -3’ |
| YTHDF1（Human） | Forward: 5’- ATACCTCACCACCTACGGACA -3’ |
|  | Reverse: 5’- GTGCTGATAGATGTTGTTCCCC -3’ |
| miR-130a-5p（Human） | Forward: 5’- **CCAGGGCTTTTCAAAAATGA** -3’ |
|  | Reverse: 5’- CAGTGCAGGGTCCGAGGT -3’ |
| MDM4（Human） | Forward: 5’- CTCAGTGTCAACATCTGACAG -3’ |
|  | Reverse: 5’- CATATGCTGCTCCTGCTGATC -3’ |
| P53（Human） | Forward: 5’- CTGAGGTTGGCTCTGACTGTACCACCATCC -3’ |
|  | Reverse: 5’- CTCATTCAGCTCTCGGAACATCTCGAAGCG -3’ |
| P21（Human） | Forward: 5’- CTTCGACTTTGTCACCGAGA -3’ |
|  | Reverse: 5’- GGTCCACATGGTCTTCCTCT -3’ |
| SNAI1（Human） | Forward: 5’- AAGATGCACATCCGAAGCCA -3’ |
|  | Reverse: 5’- CATTCGGGAGAAGGTCCGAG -3’ |
| CDH1（Human） | Forward: 5’- GGGGTCTGTCATGGAAGGTG -3’ |
|  | Reverse: 5’- CAAAATCCAAGCCCGTGGTG -3’ |
| CDH2（Human） | Forward: 5’- AGGCGTTATGTGTGTATCTTCACT -3’ |
|  | Reverse: 5’- GGAGGGATGACCCAGTCTCT -3’ |
| SPP1 | Forward: 5’- CGAGGTGATAGTGTGGTTTATGG-3’ |
|  | Reverse: 5’- GCACCATTCAACTCCTCGCTTTC -3’ |
| HKDC1 | Forward: 5’- ATCCTGGCAAGCAGAGATACG -3’ |
|  | Reverse: 5’- GACGCTCTGAAATCTGCCCT -3’ |
| GMNN | Forward: 5’- CGGGCGAGCGGAGTTAGCAG -3’ |
|  | Reverse: 5’- TGGCTGCAGCACCTCGCAAA -3’ |
| U6 （Human） | Forward: 5’- CTCGCTTCGGCAGCACA -3’ |
|  | Reverse: 5’- AACGCTTCACGAATTTGCGT -3’ |
| GAPDH（Human | Forward: 5’- GCACCGTCAAGGCTGAGAAC -3’  Reverse: 5’- GGATCTCGCTCCTGGAAGATG -3’ |

**Table S4. Western blot antibody information**

| Target nam | Manufacturer | Item number | Dilution ratio |
| --- | --- | --- | --- |
| MDM4（human） | Abcam | ab243859 | 1: 100 |
| HCV NS3 | Abcam | ab13830 | 1: 1000 |
| p53（phospho S15） | Abcam | ab223868 | 1: 5000 |
| P53（human） | Abcam | ab32049 | 1: 1000 |
| P21（human） | Abcam | ab109520 | 1: 1000 |
| SNAl1 | Proteintech | 61367 | 1: 1000 |
| E-Cadherin | Abcam | ab314063 | 1: 1000 |
| N-Cadherin | Abcam | ab76011 | 1: 1000 |
| GAPDH（human） | Abcam | ab8245 | 1: 500 |

Note:Abcam,UK
